# Comparison of incidence trends of early-onset and late-onset type 2 diabetes in the Asia-Pacific region, 1990-2021: a join point regression analysis based on the global burden of disease study 2021

**DOI:** 10.3389/fendo.2025.1466428

**Published:** 2025-02-19

**Authors:** Chenying Lin, Haohua An, Jingna Lin, Yuejuan Cao, Zhen Yang

**Affiliations:** ^1^ Tianjin Union Medical Center, Tianjin Medical University, Tianjin, China; ^2^ Department of Endocrinology, Health Management Center, Tianjin Union Medical Center, The First Affiliated Hospital of Nankai University, Tianjin, China; ^3^ Department of Clinical Laboratory, Tianjin Union Medical Center, The First Affiliated Hospital of Nankai University, Tianjin, China; ^4^ Department of Cardiology, Tianjin Union Medical Center, The First Affiliated Hospital of Nankai University, Tianjin, China; ^5^ Department of Clinical Laboratory, Tianjin Cancer Institute of Integrative Traditional Chinese and Western Medicine, Tianjin Union Medical Center, The First Affliated Hospital of Nankai University, Tianjin, China

**Keywords:** incidence, adolescents, young adults, Asia-Pacific region, type 2 diabetes

## Abstract

**Introduction:**

This study evaluated the incidence trends of early-onset (diagnosed at ages 15-39) and late-onset (diagnosed at age 40 and above) type 2 diabetes mellitus (T2DM) in the Asia-Pacific region, including the World Health Organization (WHO) South-East Asia Region (SEARO) and Western Pacific Region (WPRO), and assessed the impact of the COVID-19 pandemic.

**Methods:**

Using data from the Global Burden of Diseases Study (GBD) 2021, we analyzed trends in age-standardized incidence rate (ASIR) using Join point regression to determine annual percentage change (APC). To assess the pandemic’s impact, we calculated excess incidence for 2020 and 2021 by subtracting predicted ASIR from observed ASIR.

**Results:**

In recent years, particularly during the COVID-19 pandemic, the ASIR for early-onset T2DM in the Asia-Pacific region accelerated significantly. SEARO’s APC rose from 2.24% between 2011-2019 to 5.45% between 2019-2021. Similarly, WPRO’s APC increased from 1.71% between 1999-2017 to 5.01% between 2017-2021. In 2021, the ASIR for early-onset T2DM was 269.6 per 100,000 in WPRO and 248.4 per 100,000 in SEARO. Conversely, late-onset T2DM ASIR growth in SEARO slowed after 2017 (APC 1.92% for 2005-2017 *vs*. 1.04% for 2017-2021), while WPRO saw a decline (APC 1.06% for 2007-2017 *vs*. -1.10% for 2017-2021). During the COVID-19 pandemic in 2020 and 2021, the observed ASIR of early-onset T2DM in the Asia-Pacific region exceeded historical predictions, showing a positive excess in ASIR.

**Conclusions:**

This study reveals a significant recent increase in early-onset T2DM incidence in the Asia-Pacific region, highlighting the need for targeted public health interventions.

## 1 Introduction

Type 2 diabetes presents a dual challenge: a high prevalence burden in an aging society and an accelerating incidence among younger populations (1–3). Early-onset type 2 diabetes, defined as type 2 diabetes diagnosed before the age of 40 in adolescents and young adults (2–4), progresses more rapidly and has a higher risk of complications compared to late-onset type 2 diabetes (2, 3). Additionally, it imposes greater economic and psychological burdens on younger populations, further exacerbating the overall burden of type 2 diabetes (2, 3). According to the Global Burden of Disease Study (GBD) 2019 data, the age-standardized incidence rate (ASIR) of early-onset T2DM in the global population aged 15-39 increased from 117.2 per 100,000 people in 1990 to 183.4 per 100,000 in 2019 (4).

The Asia-Pacific region faces rapid population growth, aging populations, and the rapid development of the digital economy, all contributing to an increased type 2 diabetes burden, particularly in countries such as China, India, and Indonesia (5–8). Since the latter half of the 20th century, rapid industrialization and urbanization in the Asia-Pacific region, including the WHO’s Western Pacific Region (WPRO) and South-East Asia Region (SEARO), have significantly altered lifestyles and led to a rise in type 2 diabetes cases (5, 6). China and India, the world’s most populous countries, have the highest number of diabetes cases globally. As of 2021, China had 145.4 million people living with diabetes, followed by India with 74.2 million (6, 7), Studies have shown that the prevalence of type 2 diabetes in Asia is increasing more rapidly and affecting younger populations compared to other regions (9). Despite this, there is a lack of research specifically on early-onset type 2 diabetes in the Asia-Pacific region, and the trends of early-onset type 2 diabetes in this area, especially recent changes, have not been widely addressed.

The COVID-19 pandemic has further impacted the burden of type 2 diabetes. SARS-CoV-2 can induce new-onset type 2 diabetes through various mechanisms, including triggering inflammation and insulin resistance, disrupting glucose and lipid metabolism, and causing adipose tissue dysfunction (10–12), Lockdown measures have complicated type 2 diabetes management, and disruptions in medical services coupled with lifestyle changes, such as reduced physical activity and increased unhealthy eating, have heightened the risk of type 2 diabetes (13). Studies from Germany and the United States indicate that the incidence of type 2 diabetes among children and adolescents significantly increased during the COVID-19 pandemic (14, 15). However, there is a lack of systematic research on the potential impact of the COVID-19 pandemic on the burden of early-onset type 2 diabetes in the Asia-Pacific region.

This study aims to fill this research gap by utilizing data from the GBD 2021 to evaluate and compare the trends in the burden of early-onset and late-onset type 2 diabetes in the Asia-Pacific region from 1990 to 2021, with a particular focus on the past decade (2010 to 2021). Additionally, we aim to analyze the potential impact of the COVID-19 pandemic by calculating the excess incidence rates for 2020 and 2021, providing scientific evidence to inform effective public health policies and interventions in the Asia-Pacific region.

## 2 Subjects, materials and methods

### 2.1 Overview

This descriptive repeated cross-sectional analysis evaluates and compares incidence trends of early-onset (15-39 years) and late-onset (≥40 years) type 2 diabetes in the Asia-Pacific region, focusing on trends from 2010 to 2021 and the impact of the COVID-19 pandemic. The Asia-Pacific region includes the WHO South-East Asia Region (SEARO) and the Western Pacific Region (WPRO). SEARO comprises 11 countries: Bangladesh, Bhutan, Democratic People’s Republic of Korea, India, Indonesia, Maldives, Myanmar, Nepal, Sri Lanka, Thailand, and Timor-Leste. WPRO includes 31 countries: American Samoa, Australia, Brunei Darussalam, Cambodia, China, Cook Islands, Fiji, Guam, Japan, Kiribati, Lao People’s Democratic Republic, Malaysia, Marshall Islands, Micronesia, Mongolia, Nauru, New Zealand, Niue, Northern Mariana Islands, Palau, Papua New Guinea, Philippines, Republic of Korea, Samoa, Singapore, Solomon Islands, Tokelau, Tonga, Tuvalu, Vanuatu, and Viet Nam (**Supplementary Figure S1**).

Using data from the Global Health Data Exchange (GHDx), we extracted incidence counts, rates, and their 95% uncertainty intervals (UIs) for these regions and their respective countries, stratified by gender and 5-year age groups from 1990 to 2021 (16). This study follows the Guidelines for Accurate and Transparent Health Estimates Reporting (GATHER) (17). This study used publicly available data from GBD 2021 and did not involve human participants. It was deemed exempt by the Ethics Committee of Nankai University Affiliated Hospital.

### 2.2 Non-fatal burden estimation of type 2 diabetes

GBD 2021 diagnosed diabetes using fasting plasma glucose (FPG), glycated hemoglobin A1c (HbA1c), oral glucose tolerance test (OGTT), and postprandial glucose test (PPG), excluding random blood glucose or self-reported diabetes status. Compared to GBD 2019, the GBD 2021 model included 115 additional data sources. Data biases were adjusted using MR-BRT (Meta-Regression—Bayesian, Regularized, Trimmed) analysis, stratified by age and sex, and standardized using Out-of-Dismod crosswalks (1, 18).

GBD 2021 used the Dismod-MR 2.1 model to estimate total diabetes prevalence and incidence. For children under 15, estimates from the type 1 diabetes model replaced total diabetes estimates, assuming type 2 diabetes does not occur before age 15. Type 2 diabetes burden was estimated by subtracting type 1 diabetes estimates from total diabetes estimates. Multiple simulations with Dismod-MR 2.1 generated posterior distributions of parameters to obtain 95% UIs for disease burden indicators (1, 18). Detailed information is in **Supplementary Section 1**.

Early-onset type 2 diabetes, historically defined as diagnosed before age 40 (2, 3), is defined in this study as type 2 diabetes in individuals aged 15 to 39. Type 2 diabetes in individuals aged 40 and above is classified as late-onset type 2 diabetes.

### 2.3 Statistical analysis

To ensure comparability across regions and account for age structure differences, we calculated age-standardized rates (ASR) using the world standard population from GBD 2021 (7). This involved multiplying the age-specific rate by the standard population weight for each age group, summing these weighted rates, and dividing by the total standard population weight to obtain the ASR. ASRs for early-onset (15-39 years) and late-onset (40 years and above) type 2 diabetes were calculated separately. The calculation formula is as follows:


$$ASR=\left(\frac{\sum_{i=1}^{A}a_{i}w_{i}}{\sum_{i=1}^{A}w_{i}}\right)$$

where: α_i_ is the age-specific rate for the i-th age group (per 100,000 population), *w_i_* is the weight proportion of the i-th age group in the world standard population, and A is the total number of age groups.

To compare trends in age-standardized incidence rates (ASIR) of early-onset and late-onset type 2 diabetes across different regions from 1990 to 2021, particularly from 2010 to 2021, we used join point regression analysis. This model identifies points where significant trend changes occur and estimates the slope between these points, describing the annual percent change (APC) in incidence rates for specific periods. The average annual percent change (AAPC) is used to capture the overall trend. A positive APC or AAPC indicates an increasing trend, while a negative value indicates a decreasing trend. We calculated AAPCs for 1990-2010, and 2010-2021 to compare recent trends with previous periods. The number of join points was determined using the Grid Search Method (GSM) and validated through Monte Carlo permutation tests. Statistical significance of APC and AAPC values was assessed using 95% confidence intervals (CIs). A change is statistically significant if the CI does not include zero. To identify the potential impact of the COVID-19 pandemic, we performed join point regression analysis on all Asia-Pacific countries showing significant changes in early-onset type 2 diabetes trends after 2018.

To further explore the COVID-19 pandemic’s impact, we calculated excess incidence rates for 2020 and 2021. Excess incidence rates were defined as the observed incidence minus the expected incidence based on historical trends. We used data from 1990-2019 and employed the Bayesian Age-Period-Cohort (BAPC) model for predictions, which combines Bayesian inference with traditional Age-Period-Cohort analysis. Bayesian inference was conducted using Integrated Nested Laplace Approximations (INLA), improving computational efficiency by converting high-dimensional integrals into low-dimensional integrals. This method allowed us to predict expected incidence rates for 2020 and 2021. Excess incidence rates were then calculated by subtracting expected incidence rates from observed incidence rates, reflecting the abnormal changes during the COVID-19 pandemic. All statistical analyses were conducted using the Join point Regression Program (version 5.0.2) and R software (version 4.3.2). Differences were considered statistically significant at *P*<0.05 (two-sided).

## 3 Results

### 3.1 Comparison of early-onset and late-onset type 2 diabetes incidence trends by region

**Figure 1:**
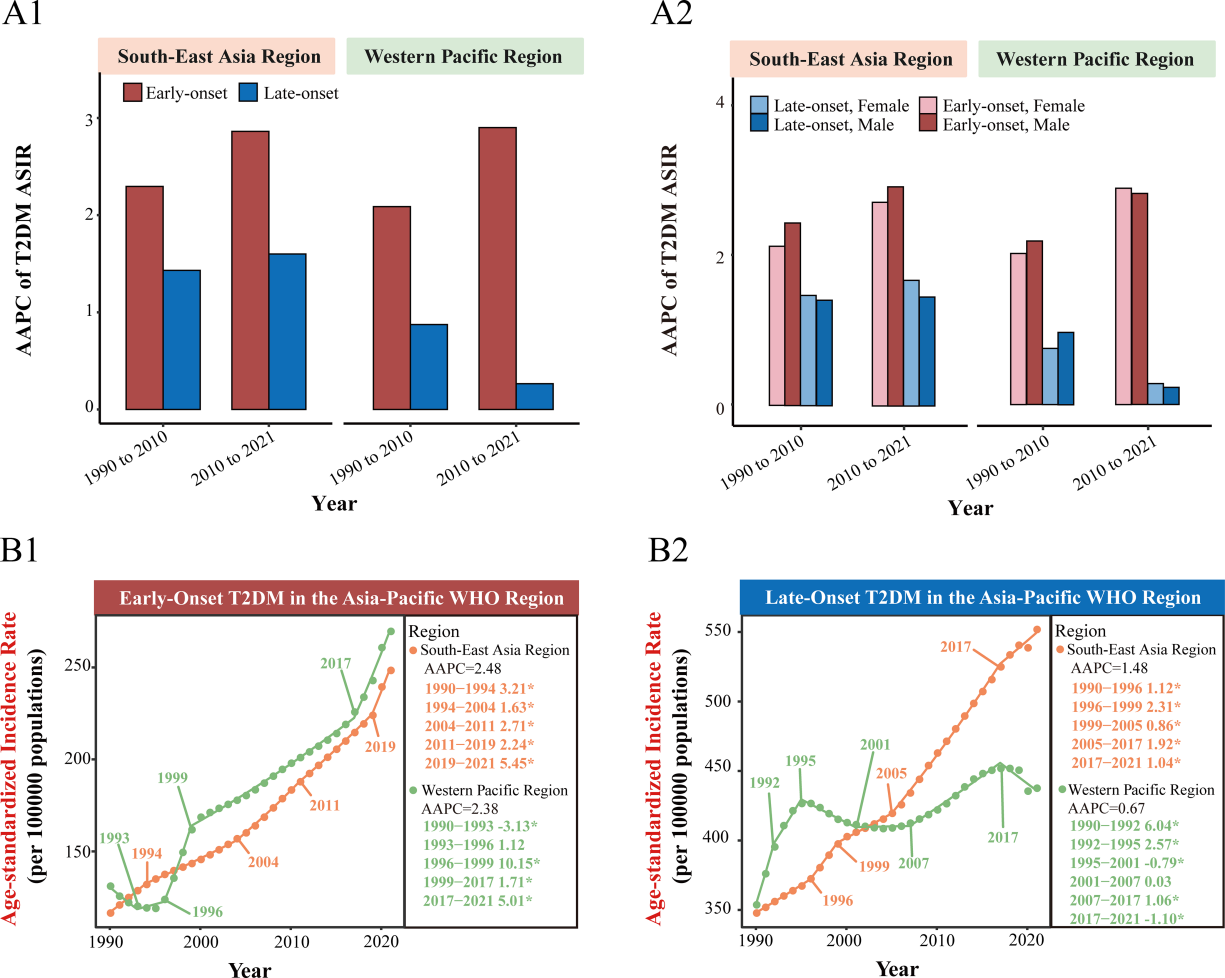
Comparison of early-onset and late-onset type 2 diabetes incidence trends in the WHO South-East Asia Region and Western Pacific Region. **(A1)** Average annual percent change (AAPC) of age-standardized incidence rates (ASIR) for early-onset and late-onset type 2 diabetes in the WHO South-East Asia Region and Western Pacific Region for the periods 1990-2010 and 2010-2021. **(A2)** AAPC of ASIR for early-onset and late-onset type 2 diabetes further stratified by sex in the WHO South-East Asia Region and Western Pacific Region for the periods 1990-2010 and 2010-2021. **(B1)** Joinpoint regression analysis of early-onset type 2 diabetes ASIR trends in the WHO South-East Asia Region and Western Pacific Region from 1990 to 2021. **(B2)** Joinpoint regression analysis of late-onset type 2 diabetes ASIR trends in the WHO South-East Asia Region and Western Pacific Region from 1990 to 2021. APC, annual percentage change. ^*^ *P*<0.05 for significant APC.

In SEARO, early-onset type 2 diabetes ASIR was slightly lower than in WPRO, at 248.4 per 100,000 (95% CI: 176.8 to 326.6) and 269.6 per 100,000 (95% CI: 194.8 to 354), respectively. However, late-onset type 2 diabetes ASIR was significantly higher in SEARO compared to WPRO, at 551.8 per 100,000 (95% CI: 421.4 to 703.4) versus 437.6 per 100,000 (95% CI: 322.5 to 570.5) (**Figure 1**; **Table 1**). Detailed incidence trends stratified by sex and by 5-year age groups are provided in **Supplementary Sections 4** and **5**.

**Table 1:** ASIR and AAPC of early-onset and late-onset type 2 diabetes by region, country, and sex from 1990 to 2021.

Location/Sex	Early-onset type 2 diabetes						Late-onset type 2 diabetes					
	ASIR (per 100,000)		AAPC				ASIR (per 100,000)		AAPC			
	1990	2021	1990-2010	P-Value^*^	2010-2021	P-Value^**^	1990	2021	1990-2010	P-Value^*^	2010-2021	P-Value^**^
South-East Asia Region	116·5 (80·8 to 157·1)	248·4 (176·8 to 326·6)	2·3 (2·24 to 2·35)	<0·001	2·86 (2·75 to 2·98)	<0·001	347·8 (259·9 to 453·7)	551·8 (421·4 to 703·4)	1·43 (1·39 to 1·47)	<0·001	1·6 (1·49 to 1·72)	<0·001
Male	123·5 (85·7 to 166·3)	273·3 (196·7 to 359)	2·44 (2·39 to 2·49)	<0·001	2·93 (2·86 to 3)	<0·001	362·3 (270·3 to 473·4)	566·3 (429·9 to 723)	1·41 (1·35 to 1·48)	<0·001	1·46 (1·31 to 1·6)	<0·001
Female	109·2 (75·4 to 147·9)	222·6 (156·5 to 294·7)	2·13 (2·06 to 2·2)	<0·001	2·72 (2·59 to 2·85)	<0·001	332·9 (250·4 to 434·8)	537·3 (411·2 to 684·5)	1·47 (1·45 to 1·5)	<0·001	1·68 (1·59 to 1·77)	<0·001
Bangladesh	132·4 (94·1 to 174·3)	326·1 (236·7 to 422·9)	2·91 (2·76 to 3·06)	<0·001	3·17 (2·87 to 3·46)	<0·001	329·2 (259·4 to 408·9)	537 (405·2 to 683·7)	1·67 (1·65 to 1·69)	<0·001	1·38 (1·27 to 1·49)	<0·001
Bhutan	113·2 (79·2 to 152·4)	217 (157 to 286)	2·17 (2·15 to 2·2)	<0·001	1·93 (1·81 to 2·05)	<0·001	288·7 (213·1 to 376·3)	478·5 (360·8 to 611·1)	1·68 (1·6 to 1·75)	<0·001	1·52 (1·38 to 1·67)	<0·001
Democratic People’s Republic of Korea	107·2 (73·2 to 145·4)	226 (163·8 to 296·9)	2·39 (2·39 to 2·4)	<0·001	2·51 (2·49 to 2·53)	<0·001	252·8 (186 to 333·5)	452·7 (334·6 to 577·4)	1·82 (1·79 to 1·84)	<0·001	2·06 (1·86 to 2·26)	<0·001
India	126·8 (87·2 to 173·1)	263·7 (186·3 to 351·2)	2·35 (2·32 to 2·38)	<0·001	2·45 (2·37 to 2·52)	<0·001	328 (235·7 to 442·5)	474·7 (344·2 to 630·6)	1·08 (1·03 to 1·12)	<0·001	1·35 (1·29 to 1·41)	<0·001
Indonesia	77·7 (51·7 to 106·6)	138 (95·7 to 184·4)	-0·26 (-0·76 to 0·24)	0·314	6·26 (2·46 to 10·2)	0·001	347·6 (255·6 to 458·5)	653·8 (494·6 to 837·5)	2·59 (2·52 to 2·66)	<0·001	0·97 (0·54 to 1·4)	<0·001
Maldives	74·3 (50·4 to 101)	132·9 (93·2 to 181·3)	1·39 (1·32 to 1·46)	<0·001	2·85 (2·72 to 2·97)	<0·001	451·6 (347·4 to 574·7)	775·5 (606·4 to 959·2)	1·58 (1·56 to 1·6)	<0·001	2·12 (2·08 to 2·15)	<0·001
Myanmar	114·2 (77·8 to 155·9)	222·2 (162·6 to 291·3)	2·32 (2·26 to 2·38)	<0·001	1·95 (1·91 to 2)	<0·001	570·7 (429·5 to 725·7)	1094·8 (865 to 1335·1)	2·1 (2·08 to 2·13)	<0·001	2·19 (2·13 to 2·25)	<0·001
Nepal	145·1 (100·4 to 197·1)	325·1 (233·7 to 429·1)	2·4 (2 to 2·8)	<0·001	3·15 (2·92 to 3·38)	<0·001	313·3 (228·3 to 411·6)	534·7 (389·7 to 701·1)	1·87 (1·82 to 1·91)	<0·001	1·49 (1·18 to 1·81)	<0·001
Sri Lanka	106·2 (70·7 to 148·4)	270·9 (187·5 to 360)	4·13 (4·03 to 4·22)	<0·001	1·16 (1·06 to 1·26)	<0·001	731·7 (564·3 to 917·7)	1412 (1104·6 to 1728·4)	2·43 (2·39 to 2·46)	<0·001	1·64 (1·5 to 1·77)	<0·001
Thailand	73·3 (50·3 to 99)	149·2 (105·4 to 198·1)	2·56 (2·5 to 2·61)	<0·001	2·04 (1·77 to 2·32)	<0·001	395·9 (302·7 to 498·6)	740·8 (579·8 to 912·5)	1·78 (1·76 to 1·8)	<0·001	2·48 (2·41 to 2·55)	<0·001
Timor-Leste	51·1 (35·8 to 68·2)	151·7 (108·2 to 201·2)	3·4 (3·37 to 3·43)	<0·001	3·92 (3·83 to 4·01)	<0·001	298 (233·9 to 371·1)	837·2 (645·6 to 1056·3)	3·4 (3·35 to 3·45)	<0·001	3·46 (3·42 to 3·51)	<0·001
Western Pacific Region	131·1 (85·4 to 187·5)	269·6 (194·8 to 354)	2·09 (1·7 to 2·47)	<0·001	2·9 (2·72 to 3·08)	<0·001	353·7 (251 to 475·5)	437·6 (322·5 to 570·5)	0·87 (0·81 to 0·94)	<0·001	0·27 (0·09 to 0·44)	0·003
Male	148·5 (96·8 to 212·2)	307·7 (223·7 to 402·4)	2·18 (1·89 to 2·49)	<0·001	2·82 (2·64 to 3)	<0·001	344·6 (243 to 467·6)	432·6 (315·6 to 569·5)	0·97 (0·91 to 1·02)	<0·001	0·23 (0·05 to 0·4)	0·011
Female	112·9 (72·9 to 162·3)	228 (162·4 to 303·3)	2·02 (1·74 to 2·3)	<0·001	2·89 (2·71 to 3·08)	<0·001	364 (259·5 to 485·3)	442·2 (326·8 to 573·5)	0·75 (0·58 to 0·92)	<0·001	0·28 (0·13 to 0·43)	<0·001
American Samoa	376·6 (265·1 to 504·1)	1022·5 (720·6 to 1376·1)	4·05 (3·98 to 4·11)	<0·001	1·82 (1·65 to 1·98)	<0·001	921·8 (684·9 to 1189·7)	1423·5 (1037·4 to 1835)	1·63 (1·6 to 1·67)	<0·001	1·04 (1 to 1·09)	<0·001
Australia	44·6 (30·4 to 62·7)	84·4 (56·4 to 121·4)	2·44 (2·42 to 2·46)	<0·001	1·43 (1·35 to 1·5)	<0·001	262·8 (203·4 to 321·6)	456·8 (346 to 568·5)	1·82 (1·78 to 1·86)	<0·001	1·74 (1·67 to 1·8)	<0·001
Brunei Darussalam	149·4 (108·1 to 197·6)	475·5 (331·7 to 646·2)	3·43 (3·36 to 3·49)	<0·001	4·46 (4·37 to 4·55)	<0·001	924·9 (743·6 to 1134·1)	1483·8 (1124·5 to 1854·8)	1·2 (1·14 to 1·25)	<0·001	2·19 (2·1 to 2·28)	<0·001
Cambodia	58·2 (39·7 to 77·8)	130·1 (92·8 to 171·3)	2·41 (2·36 to 2·45)	<0·001	3·04 (2·9 to 3·18)	<0·001	319·6 (249·2 to 395·8)	732 (572·4 to 907·4)	2·69 (2·67 to 2·71)	<0·001	2·77 (2·68 to 2·87)	<0·001
China	140·1 (89·1 to 204·5)	316·1 (226·8 to 417·7)	2·26 (1·55 to 2·97)	<0·001	3·36 (3·17 to 3·55)	<0·001	324 (214·4 to 454·4)	365·4 (251·1 to 499·1)	0·79 (0·69 to 0·88)	<0·001	-0·38 (-0·61 to -0·15)	0·001
Cook Islands	418·9 (293·7 to 559·7)	897·7 (641·7 to 1191·6)	3·26 (3·18 to 3·34)	<0·001	1·13 (0·99 to 1·27)	<0·001	1059 (770·1 to 1379·2)	1394·5 (1048·2 to 1758)	0·8 (0·77 to 0·83)	<0·001	1·04 (0·96 to 1·13)	<0·001
Fiji	276·6 (196·6 to 365·7)	585·2 (429·8 to 755)	2·05 (2·03 to 2·08)	<0·001	3·21 (3·13 to 3·28)	<0·001	1199·7 (934·8 to 1468·6)	1814·7 (1435·4 to 2225·3)	1·91 (1·84 to 1·99)	<0·001	0·28 (0·24 to 0·32)	<0·001
Guam	189·3 (136 to 250·6)	393·9 (290·5 to 512·9)	2·26 (2·23 to 2·29)	<0·001	2·62 (2·5 to 2·74)	<0·001	485·8 (371·8 to 617·6)	647·4 (497·7 to 819·5)	0·46 (0·44 to 0·48)	<0·001	1·77 (1·7 to 1·84)	<0·001
Japan	105·8 (72·4 to 144·5)	200·2 (138·2 to 274·5)	1·86 (1·81 to 1·91)	<0·001	2·46 (2·2 to 2·71)	<0·001	438·4 (331·5 to 564·7)	606·7 (449·8 to 790·5)	0·79 (0·73 to 0·85)	<0·001	1·58 (1·37 to 1·79)	<0·001
Kiribati	340·9 (244·4 to 450·7)	667 (490·5 to 860·9)	2·54 (2·5 to 2·59)	<0·001	1·55 (1·49 to 1·62)	<0·001	879·1 (664·2 to 1109·2)	1298·8 (998·6 to 1646·2)	1·82 (1·78 to 1·86)	<0·001	0·3 (0·2 to 0·41)	<0·001
Lao People’s Democratic Republic	79·4 (55·5 to 107·4)	162·8 (116·6 to 213·3)	2·14 (2·11 to 2·16)	<0·001	2·72 (2·67 to 2·77)	<0·001	415·1 (319·1 to 519·4)	862·8 (672·2 to 1065·2)	2·42 (2·41 to 2·43)	<0·001	2·34 (2·32 to 2·35)	<0·001
Malaysia	108·9 (73 to 150·2)	186 (129·1 to 252·5)	2·72 (2·68 to 2·76)	<0·001	0·04 (-0·54 to 0·62)	0·900	598·8 (460·9 to 742)	1004·3 (784·9 to 1247·2)	1·37 (1·33 to 1·41)	<0·001	2·19 (1·86 to 2·52)	<0·001
Marshall Islands	462·9 (319·9 to 621)	1136·4 (810·2 to 1521·6)	3·67 (3·49 to 3·84)	<0·001	1·58 (1·42 to 1·75)	<0·001	924·3 (665·9 to 1230·9)	1337·8 (970·2 to 1738·7)	1·43 (1·37 to 1·48)	<0·001	0·81 (0·79 to 0·82)	<0·001
Micronesia (Federated States of)	283·7 (203·7 to 370·8)	625·4 (454·9 to 820·2)	3·95 (3·86 to 4·03)	<0·001	0·3 (-0·26 to 0·87)	0·288	730·5 (557·1 to 929·5)	1270·4 (973·7 to 1588·2)	2·19 (2·17 to 2·22)	<0·001	1·05 (0·95 to 1·14)	<0·001
Mongolia	64 (43·8 to 85·6)	180 (130·4 to 235·4)	3·51 (3·45 to 3·57)	<0·001	3·22 (3·14 to 3·29)	<0·001	152·9 (115·1 to 196·6)	304·8 (227·6 to 394·1)	1·87 (1·82 to 1·91)	<0·001	2·92 (2·8 to 3·04)	<0·001
Nauru	345·1 (241·8 to 457)	728·6 (535·1 to 955·8)	2·92 (2·85 to 2·99)	<0·001	1·57 (1·43 to 1·71)	<0·001	879·3 (651·7 to 1118)	1305·6 (966·1 to 1673·8)	1·71 (1·65 to 1·78)	<0·001	0·55 (0·46 to 0·65)	<0·001
New Zealand	66·4 (38·8 to 97·3)	124·1 (90 to 159·7)	0·67 (0·57 to 0·77)	<0·001	4·66 (4·62 to 4·71)	<0·001	367·9 (270·8 to 483·9)	510·4 (416·5 to 606·9)	0·37 (0·31 to 0·44)	<0·001	2·4 (2·35 to 2·44)	<0·001
Niue	353·8 (249·7 to 471·7)	866·7 (628·3 to 1145·1)	3·25 (3·2 to 3·29)	<0·001	2·3 (2·25 to 2·34)	<0·001	838·8 (618·2 to 1087·2)	1315·6 (945·1 to 1714·6)	1·66 (1·64 to 1·69)	<0·001	1·1 (1·05 to 1·14)	<0·001
Northern Mariana Islands	206·2 (147 to 271)	432·8 (318·4 to 564·6)	2·46 (2·43 to 2·48)	<0·001	2·31 (2·28 to 2·35)	<0·001	507·2 (386·9 to 650·1)	792·6 (602·6 to 997·9)	1·48 (1·45 to 1·5)	<0·001	1·39 (1·36 to 1·41)	<0·001
Palau	349·9 (247 to 465·9)	826 (599·1 to 1078)	3·04 (3 to 3·07)	<0·001	2·4 (2·34 to 2·46)	<0·001	897·5 (663·4 to 1154·1)	1271·5 (950·6 to 1601·9)	1·35 (1·31 to 1·39)	<0·001	0·71 (0·68 to 0·73)	<0·001
Papua New Guinea	244·1 (176·1 to 320·5)	542·2 (399·9 to 697·2)	2·57 (2·54 to 2·61)	<0·001	2·7 (2·63 to 2·77)	<0·001	622·1 (475·6 to 782·3)	990·9 (753·6 to 1236·5)	1·98 (1·95 to 2)	<0·001	0·67 (0·48 to 0·86)	<0·001
Philippines	82·1 (54·1 to 113·6)	109·4 (75·8 to 148·5)	-0·38 (-0·5 to -0·27)	<0·001	3·12 (2·91 to 3·33)	<0·001	534·6 (405·4 to 684·6)	660·9 (506·1 to 837·4)	0·51 (0·47 to 0·55)	<0·001	0·98 (0·86 to 1·11)	<0·001
Republic of Korea	145·7 (106·5 to 189·6)	398·1 (289·6 to 514·7)	2·99 (2·78 to 3·2)	<0·001	3·87 (3·75 to 3·99)	<0·001	467·6 (359·6 to 574·5)	931·1 (735·3 to 1143)	2·2 (2·16 to 2·24)	<0·001	2·36 (2·34 to 2·38)	<0·001
Samoa	315·1 (222·2 to 420·7)	752·4 (546·7 to 998·6)	3·45 (3·4 to 3·49)	<0·001	1·74 (1·68 to 1·81)	<0·001	787·9 (590·1 to 1014·3)	1138·1 (841·2 to 1470·9)	1·29 (1·27 to 1·31)	<0·001	1·03 (0·97 to 1·09)	<0·001
Singapore	187·7 (129·4 to 254·7)	315·5 (223·9 to 423·5)	1·17 (1·11 to 1·23)	<0·001	2·61 (2·52 to 2·71)	<0·001	626·7 (485·1 to 781)	734 (565·2 to 918·5)	0·48 (0·46 to 0·51)	<0·001	0·59 (0·51 to 0·66)	<0·001
Solomon Islands	198·5 (144·9 to 258·4)	423·4 (320·1 to 539·4)	2·63 (2·57 to 2·69)	<0·001	2·19 (2·12 to 2·27)	<0·001	530·9 (416·8 to 662·7)	929·5 (743·7 to 1129·8)	2·33 (2·25 to 2·4)	<0·001	0·91 (0·85 to 0·97)	<0·001
Tokelau	392·3 (276·4 to 529·6)	828·5 (600 to 1101·1)	2·79 (2·74 to 2·85)	<0·001	1·79 (1·67 to 1·91)	<0·001	857·2 (622·8 to 1143·3)	1267·7 (943·2 to 1620·3)	1·53 (1·49 to 1·57)	<0·001	0·79 (0·77 to 0·82)	<0·001
Tonga	273·1 (192·2 to 365·3)	588·8 (423·7 to 776·7)	2·61 (2·59 to 2·64)	<0·001	2·3 (2·27 to 2·33)	<0·001	807·9 (611·1 to 1029·7)	1203·6 (920·3 to 1520·2)	1·36 (1·31 to 1·4)	<0·001	1·22 (1·13 to 1·31)	<0·001
Tuvalu	221 (158·9 to 291·4)	497·2 (365·3 to 645·6)	2·66 (2·63 to 2·68)	<0·001	2·58 (2·55 to 2·61)	<0·001	665·3 (507·5 to 840·8)	1016 (777 to 1270·7)	1·77 (1·73 to 1·81)	<0·001	0·65 (0·61 to 0·7)	<0·001
Vanuatu	205·1 (146·3 to 272·5)	524·5 (385·7 to 678)	3·06 (3·04 to 3·09)	<0·001	3·05 (3·01 to 3·08)	<0·001	531·4 (402·8 to 679·6)	893·2 (678·4 to 1128·5)	1·77 (1·72 to 1·83)	<0·001	1·55 (1·49 to 1·6)	<0·001
Viet Nam	57·9 (39·7 to 78)	104·3 (75·5 to 136·4)	1·07 (1·05 to 1·08)	<0·001	3·22 (3 to 3·44)	<0·001	382 (294·4 to 473·6)	715 (581·5 to 862·1)	2·03 (2 to 2·06)	<0·001	2·04 (1·98 to 2·1)	<0·001

ASIR, age-standardized incidence rates; AAPC, average annual percent change. Data in parentheses are 95% confidence intervals (CIs) for ASIR and AAPC. ^*^ *P* value for AAPC from 1990 to 2010. ^**^ *P* value for AAPC from 2010 to 2021.

Across SEARO and WPRO, the AAPC for early-onset type 2 diabetes ASIR was higher than for late-onset type 2 diabetes in both 1990-2010 and 2010-2021. In SEARO, the AAPC for early-onset type 2 diabetes ASIR was 2.3% (95% CI: 2.24% to 2.35%) from 1990-2010, and 2.86% (95% CI: 2.75% to 2.98%) from 2010-2021. In WPRO, it was 2.09% (95% CI: 1.7% to 2.47%) from 1990-2010, and 2.9% (95% CI: 2.72% to 3.08%) from 2010-2021. Join point regression analysis revealed an accelerated increase in early-onset type 2 diabetes ASIR during the COVID-19 pandemic. In SEARO, early-onset type 2 diabetes ASIR had an APC of 5.45% during 2019-2021, while WPRO had an APC of 5.01% during 2017-2021, significantly higher than the growth rate from 1999-2017 (APC 1.71%) (**Figure 1**; **Table 1**).

Conversely, late-onset type 2 diabetes ASIR in the Asia-Pacific region showed a declining or decelerating trend after 2017. In SEARO, the AAPC for late-onset type 2 diabetes ASIR was 1.43% from 1990-2010 and 1.6% from 2010-2021. Join point analysis showed a deceleration after 2017 (2005-2017 APC 1.92%; 2017-2021 APC 1.04%). In WPRO, the AAPC for late-onset type 2 diabetes ASIR was 0.87% from 1990-2010 and 0.27% from 2010-2021. Join point regression analysis indicated a reversal in the trend for late-onset type 2 diabetes ASIR in WPRO after 2017, with a significant decline (2007-2017 APC 1.06%; 2017-2021 APC -1.10%) (**Figure 1**; **Table 1**).

### 3.2 Excess incidence due to the COVID-19 pandemic across age groups and regions

**Figure 2:**
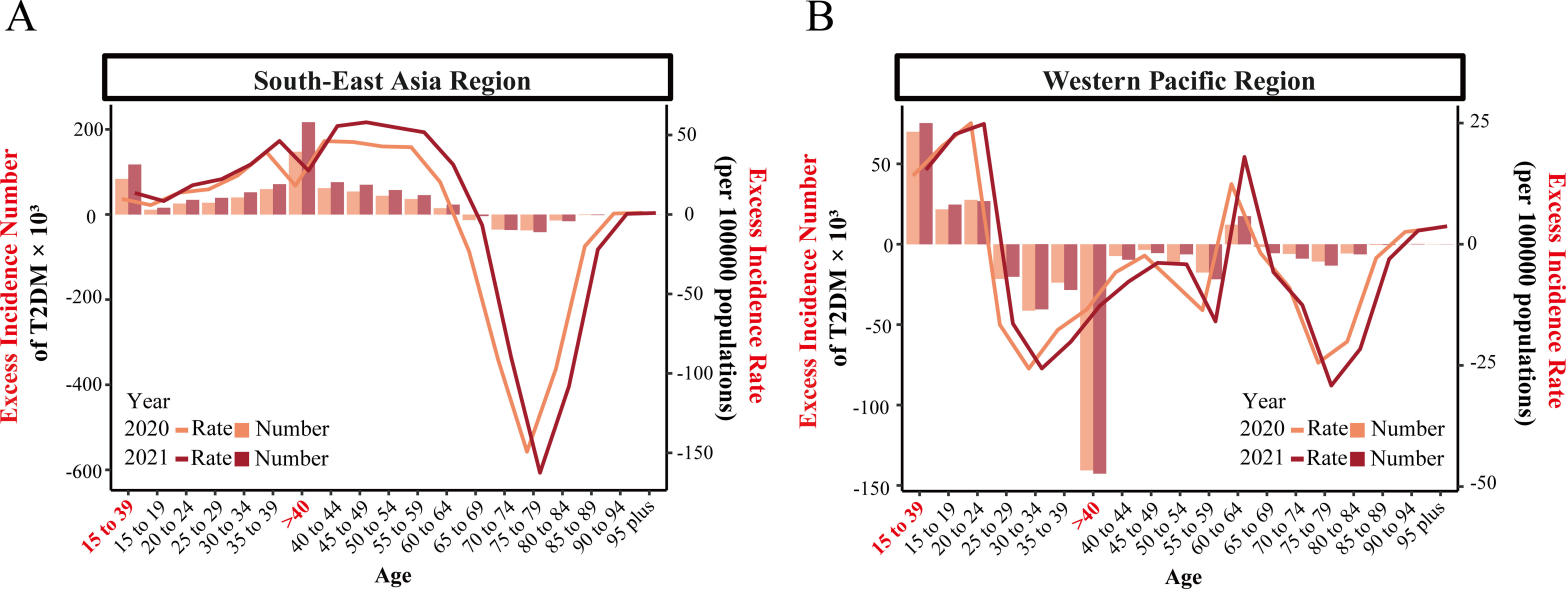
Net excess incidence number and rate of early-onset and late-onset type 2 diabetes by 5-year age groups in SEARO and WPRO for 2020 and 2021. **(A)** Net excess incidence number and rate in the South-East Asia Region (SEARO). **(B)** Net excess incidence number and rate in the Western Pacific Region (WPRO). Excess incidence numbers are represented by bars, and excess incidence rates are depicted by lines.

For early-onset type 2 diabetes, the actual incidence rates in the Asia-Pacific region were higher than predicted for both 2020 and 2021. In SEARO, COVID-19 resulted in 83,599 excess new cases in 2020, with an excess ASIR of 9.8 per 100,000. In 2021, excess new cases increased to 117,344, with an excess ASIR of 13.6 per 100,000. All 5-year age groups showed a positive excess incidence. In WPRO, there were 69,931 excess new cases in 2020, with an excess ASIR of 14.2 per 100,000, and 75,300 excess cases in 2021, with an excess ASIR of 15.3 per 100,000. However, only the 15-24 age group showed a positive excess incidence, while the 25-39 age group had a negative excess incidence (**Figure 2**; **Supplementary Table S2**).

For late-onset type 2 diabetes in SEARO, there was a positive excess incidence in both 2020 and 2021 due to COVID-19. There were 147,274 excess new cases in 2020, with an excess ASIR of 18.1 per 100,000, and 216,657 excess cases in 2021, with an excess ASIR of 27.6 per 100,000. However, the 65-89 age group showed a negative excess incidence. Conversely, in WPRO, the actual incidence of late-onset type 2 diabetes was lower than predicted in both 2020 and 2021, showing a negative excess ASIR. There were -140,650 fewer cases in 2020, with a negative excess ASIR of -13.5 per 100,000, and -142,633 fewer cases in 2021, with a negative excess ASIR of -12.7 per 100,000. All 5-year age groups in WPRO, except the 60-64 age group, showed a negative excess incidence (**Figure 2**; **Supplementary Table S2**).

### 3.3 Incidence trends of early-onset and late-onset type 2 diabetes in India and China

**Figure 3:**
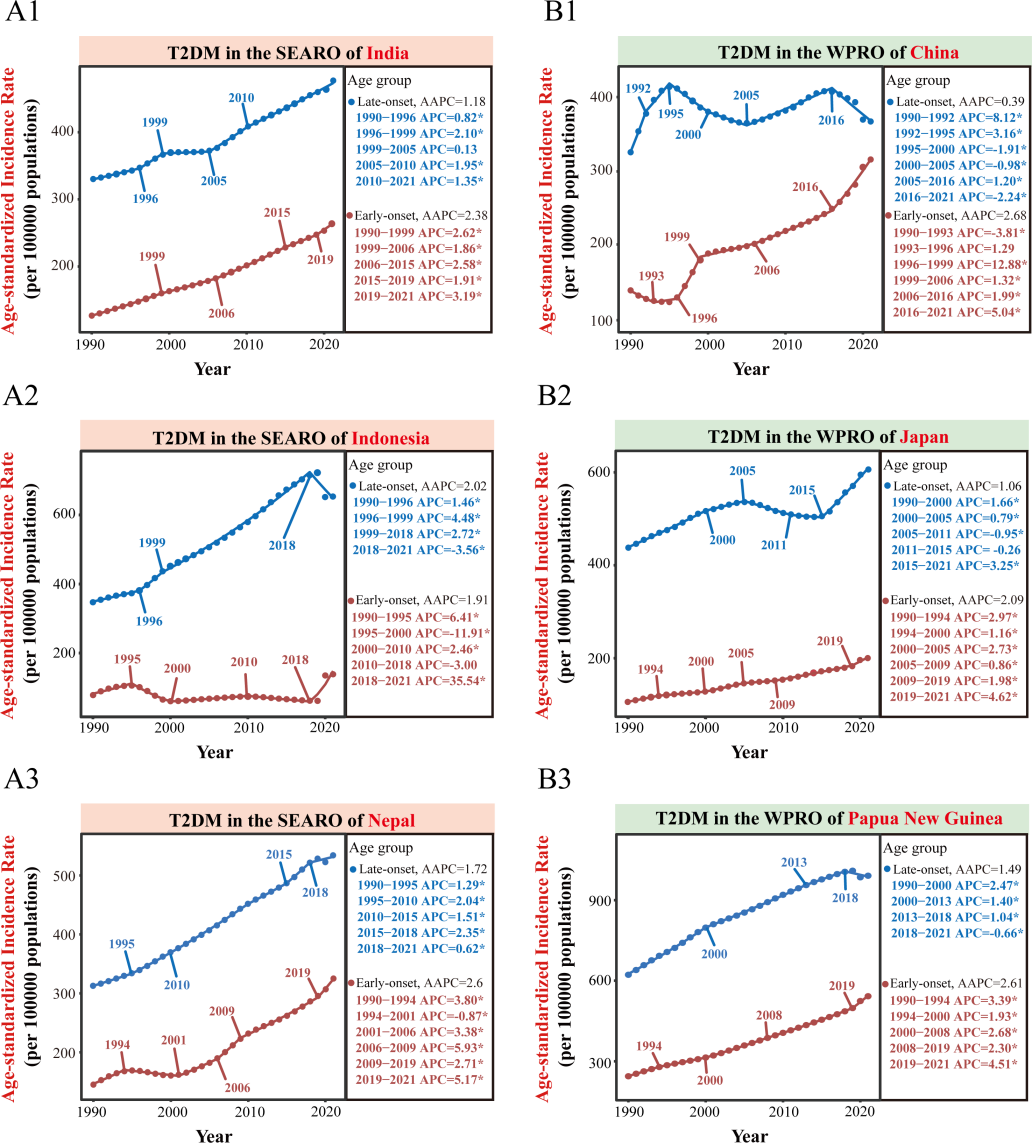
Trends in ASIR of early-onset and late-onset type 2 diabetes in selected countries from 1990 to 2021. This Figure presents the age-standardized incidence rate (ASIR) trends of early-onset and late-onset type 2 diabetes from 1990 to 2021 for selected countries in the WHO South-East Asia Region (SEARO) and Western Pacific Region (WPRO). **(A1-A3)** show the trends for SEARO’s most populous country (India) and the two countries with the fastest acceleration of early-onset type 2 diabetes during the COVID-19 pandemic (Indonesia and Nepal). **(B1-B3)** depict the trends for WPRO’s most populous country (China) and the two countries with the fastest acceleration of early-onset type 2 diabetes during the COVID-19 pandemic (Japan and Papua New Guinea). AAPC, average annual percent change. APC, annual percentage change. ^*^ *P*<0.05 for significant APC.

**Figure 4:**
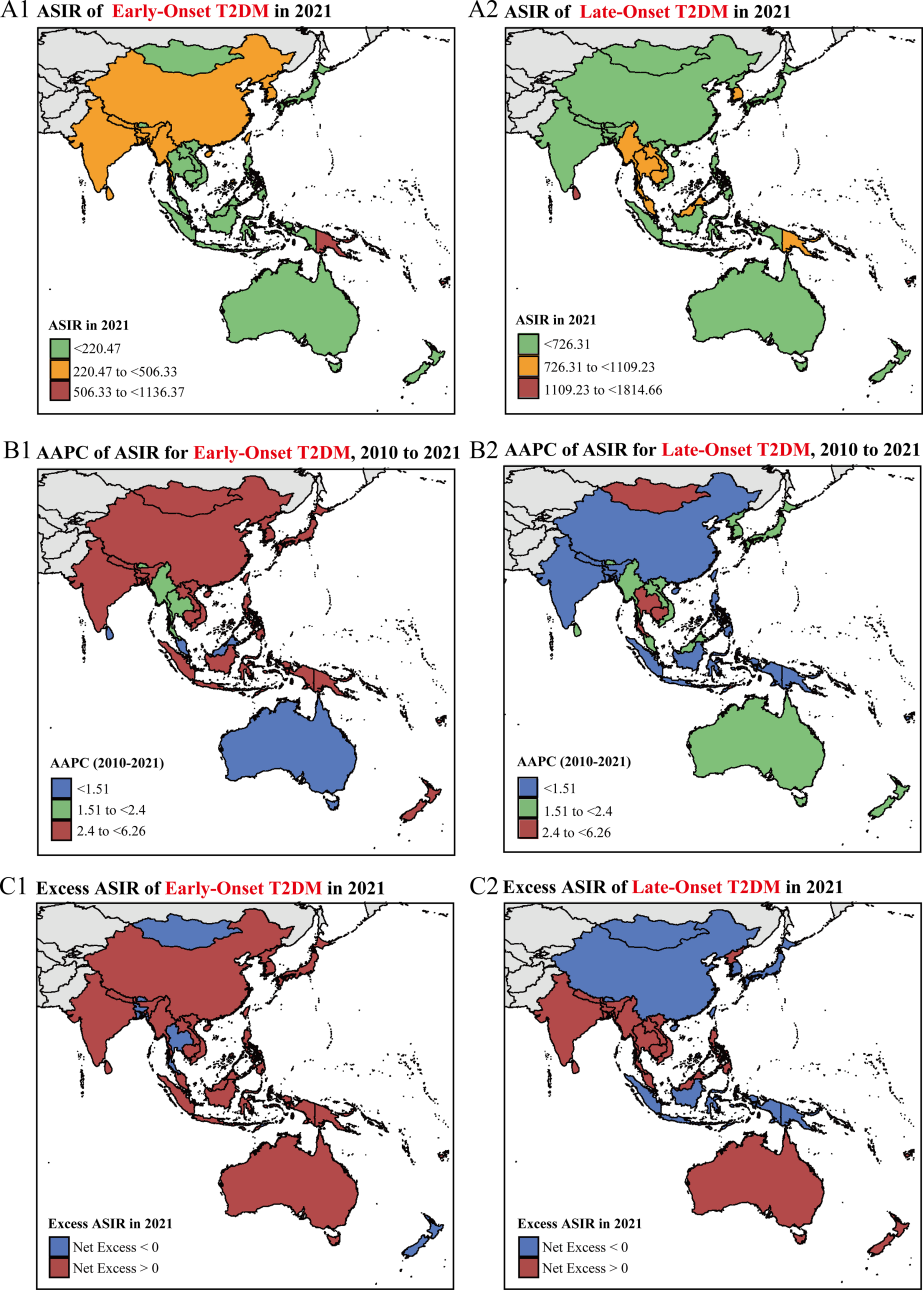
National-level ASIR trends and net excess ASIR of early-onset and late-onset type 2 diabetes in the Asia-Pacific region. **(A1-A2)** Age-standardized incidence rates (ASIR) per 100,000 people of early-onset **(A1)** and late-onset **(A2)** type 2 diabetes in 2021. **(B1-B2)** Average annual percentage change (AAPC) of ASIR from 2010 to 2021 for early-onset **(B1)** and late-onset **(B2)** type 2 diabetes. **(C1-C2)** Net excess ASIR due to the COVID-19 pandemic in 2021 for early-onset **(C1)** and late-onset **(C2)** type 2 diabetes.

India and China are the most populous countries in SEARO and WPRO, respectively, significantly impacting their regions’ disease burdens. From 1990 to 2021, India and China consistently accounted for the highest proportion of new early-onset type 2 diabetes cases in SEARO and WPRO, respectively, making up approximately 75% or more of the total cases (**Supplementary Figure S2**; **Supplementary Table S1**). In 2021, the number of new early-onset type 2 diabetes cases was 1.59 million (1.13 to 2.13 million) in India and 1.31 million (0.92 to 1.77 million) in China (**Supplementary Table S1**). Trends in early-onset and late-onset type 2 diabetes in both countries align closely with regional trends. In 2021, China’s ASIR for early-onset type 2 diabetes was 316.1 per 100,000 (95% CI: 226.8 to 417.7), higher than India’s 263.7 per 100,000 (95% CI: 186.3 to 351.2). Conversely, India’s ASIR for late-onset type 2 diabetes exceeded China’s, with rates of 474.7 per 100,000 (95% CI: 344.2 to 630.6) compared to China’s 365.4 per 100,000 (95% CI: 251.1 to 499.1) (**Figures 3**, **4**; **Table 1**).

In India, ASIR trends for both early-onset and late-onset type 2 diabetes show consistent annual increases, with higher growth rates from 2010 to 2021 compared to 1990 to 2010. The AAPC for early-onset type 2 diabetes from 2010 to 2021 is 2.45% (95% CI: 2.37% to 2.52%), while for late-onset type 2 diabetes it is 1.35% (95% CI: 1.29% to 1.41%). Join point regression analysis indicates a significant acceleration in early-onset type 2 diabetes during the COVID-19 pandemic (2019-2021 APC: 3.19%) compared to 1990 to 2019 (APC: 1.91% to 2.62%) (**Figures 3**, **4**; **Table 1**).

In contrast, China shows opposite trends for early-onset and late-onset type 2 diabetes. The ASIR for early-onset type 2 diabetes has a higher growth rate in the last decade compared to the previous two decades (1990-2010 AAPC: 2.26%; 2010-2021 AAPC: 3.36%; both P<0.001). Conversely, the ASIR for late-onset type 2 diabetes shows an overall upward trend from 1990 to 2010 (AAPC: 0.79%; 95% CI: 0.69% to 0.88%) but reversed in the recent decade (2010-2021 AAPC: -0.38%; 95% CI: -0.61% to -0.15%), showing a decline. Further join point analysis shows that since 2016, the ASIR for early-onset type 2 diabetes accelerated (2016-2021 APC 5.04%), while the ASIR for late-onset type 2 diabetes significantly declined (2016-2021 APC -2.24%) (**Figures 3**, **4**; **Table 1**). During the COVID-19 pandemic, early-onset type 2 diabetes ASIR exceeded expectations, with a positive excess ASIR of 19.0 per 100,000 in 2020 and 18.8 per 100,000 in 2021. In contrast, late-onset type 2 diabetes showed a negative excess ASIR, with -9.1 per 100,000 in 2020 and -7.5 per 100,000 in 2021 (**Figure 4**; **Supplementary Table S3**).

### 3.4 Incidence trends of early-onset and late-onset type 2 diabetes in other countries

In 2021, among the 11 countries in the WHO SEARO, Bangladesh had the highest ASIR of early-onset type 2 diabetes at 326.1 cases per 100,000 people (95% CI: 236.7 to 422.9), while Sri Lanka had the highest ASIR for late-onset type 2 diabetes at 1412 cases per 100,000 people (95% CI: 1104.6 to 1728.4). In the WHO WPRO, among 31 countries, the Marshall Islands had the highest ASIR for early-onset type 2 diabetes at 1136.4 cases per 100,000 people (95% CI: 810.2 to 1521.6), and Fiji had the highest ASIR for late-onset type 2 diabetes at 1814.7 cases per 100,000 people (95% CI: 1435.4 to 2225.3) (**Figure 4**; **Table 1**).

From 2010 to 2021, Indonesia experienced the fastest increase in early-onset type 2 diabetes incidence in SEARO, with an AAPC of 6.26% (95% CI: 2.46% to 10.2%). During the COVID-19 pandemic (post-2018), countries including Indonesia and Nepal showed significant accelerations in early-onset type 2 diabetes incidence. Indonesia, in particular, reversed its earlier downward trend (2010-2018 APC -3.0%) to a sharp increase from 2018-2021 (APC 35.54%). Meanwhile, Indonesia’s late-onset type 2 diabetes incidence, which had been rising steadily from 1990 to 2018 (APC 1.46% to 2.71%), declined sharply from 2018 to 2021 (APC -3.56%). Nepal and Sri Lanka also saw decelerations in late-onset type 2 diabetes ASIR during this period (**Figures 3**, **4**; **Table 1**; **Supplementary Figure S3A**). In the Asia-Pacific region, Indonesia had the highest excess ASIRs of early-onset type 2 diabetes due to COVID-19, with 74.5 per 100,000 in 2020 and 78.6 per 100,000 in 2021. Similar to trends in China, Indonesia also experienced negative excess ASIRs for late-onset type 2 diabetes, with -12.5 per 100,000 in 2020 and -13.8 per 100,000 in 2021. (**Figure 4**; **Supplementary Table S3**). From 2010 to 2021, New Zealand had the fastest increase in early-onset type 2 diabetes incidence in WPRO, with an AAPC of 4.66% (95% CI: 4.62% to 4.71%). During the COVID-19 pandemic, Japan showed the most significant acceleration in early-onset type 2 diabetes, with an APC of 4.62% from 2019 to 2021 (**Figures 3**, **4**; **Table 1**).

## 4 Discussion

In this study, we utilized data from GBD 2021 to examine the trends in early-onset and late-onset type 2 diabetes incidence in the Asia-Pacific region over the past three decades. From 1990 to 2021, early-onset type 2 diabetes incidence consistently increased, with a significant acceleration in the past decade and during the COVID-19 pandemic. Conversely, while late-onset type 2 diabetes incidence also increased over the past thirty years, the growth rate slowed in the SEARO region and declined in the WPRO region since 2017. In 2020 and 2021, the actual incidence of early-onset type 2 diabetes exceeded historical predictions, showing a positive excess incidence. For late-onset type 2 diabetes, SEARO experienced a positive excess incidence, while WPRO exhibited a negative excess incidence.

### 4.1 Comparative trends in early-onset T2DM between the Asia-Pacific region and Western countries

In recent years, the incidence of T2DM in children and adolescents has garnered increasing attention, but systematic research has primarily focused on Western countries. For instance, the SEARCH for Diabetes in Youth (SEARCH) and the Treatment Options for Type 2 Diabetes in Adolescents and Youth (TODAY) studies in the United States provide extensive evidence on T2DM in young people. The latest SEARCH report shows a significant increase in T2DM incidence among individuals under 20 from 2002 to 2018, with an annual growth rate of 5.31%. In 2017-2018, the annual incidence rate was 17.9 per 100,000, peaking at age 16 (19).

In contrast, early-onset T2DM trends in the Asia-Pacific region have not been widely studied, particularly during the COVID-19 pandemic. This study fills that gap using GBD 2021 data. Our findings show that from 1990 to 2021, early-onset T2DM incidence in the Asia-Pacific region consistently increased, with notable acceleration during the COVID-19 pandemic. The WPRO region saw a growth rate of 5.01% from 2017-2021, and the SEARO region saw a 5.45% growth from 2019-2021. Notably, incidence among youths (15-24 years) in the WPRO region consistently showed a secondary peak, higher than that of young adults (25-39 years). Additionally, during the COVID-19 pandemic in 2020 and 2021, the actual incidence rate of early-onset T2DM in the Asia-Pacific region exceeded historical predictions, indicating a positive excess incidence. This trend aligns with reports from Western countries. For example, a retrospective study in a US pediatric hospital and another study involving 24 centers found that the incidence of T2DM among adolescents increased by 182% and 77.2%, respectively, during the COVID-19 pandemic compared to pre-pandemic levels (15, 20). Similarly, a study using data from the German Diabetes Prospective Follow-up Registry reported that the actual T2DM incidence among adolescents in 2021 was 1.95 per 100,000 person-years, compared to a predicted rate of 1.38 per 100,000 person-years, indicating an incidence rate 1.41 times higher than predicted (14).

### 4.2 Potential impact of COVID-19 on the differences in early-onset and late-onset type 2 diabetes incidence trends

This study is the first to compare early-onset and late-onset type 2 diabetes incidence trends in the Asia-Pacific region. Our findings indicate that, particularly during the COVID-19 pandemic, early-onset type 2 diabetes incidence has accelerated, while late-onset type 2 diabetes incidence has slowed or declined. The COVID-19 pandemic may have both direct and indirect effects on these trends.

Numerous studies show that COVID-19 infection increases the risk of type 2 diabetes, especially among younger individuals (21, 22). A nationwide cohort study in South Korea found that COVID-19 patients had a significantly higher risk of newly diagnosed type 2 diabetes, with younger individuals showing a higher adjusted hazard ratio (HR 1.54) than older individuals (HR 1.19) (21). Additionally, COVID-19 infection may accelerate the progression from prediabetes to new-onset diabetes (23), especially in younger populations. Younger prediabetic individuals are more likely to progress to type 2 diabetes than older individuals (24), and a cohort study indicated that older prediabetic patients are more likely to return to normoglycemia or die rather than progress to diabetes during the observation period (25). SARS-CoV-2 may cause insulin resistance and β-cell damage through mechanisms like binding to ACE2 receptors, inducing cytokine storms, and causing adipose tissue inflammation (10, 11). Additionally, the high mortality rate among older adults due to COVID-19 may statistically lower the diabetes incidence in this age group, contributing to the divergent trends (26).

The pandemic may also indirectly affect type 2 diabetes risk, especially among younger individuals. Lockdown measures have increased negative health behaviors, such as greater food intake, junk food consumption, reduced physical activity, and increased screen time. Younger people are more prone to these behaviors than older individuals (27, 28). These lifestyle changes have led to significant weight gain (28). A nationwide retrospective cohort study in China found that the impact of BMI on diabetes incidence is more pronounced in younger adults, with the association between BMI and diabetes risk weakening with age (29).

### 4.3 China and India’s unique challenges and characteristics in the burden of type 2 diabetes

China and India, the world’s most populous countries, significantly impact type 2 diabetes trends in their regions, but their trends differ markedly. From 1990 to 2021, both early-onset and late-onset type 2 diabetes ASIR in India showed a steady upward trend, with early-onset type 2 diabetes incidence accelerating post-2019 during the COVID-19 pandemic. Conversely, China saw an accelerating trend in early-onset type 2 diabetes even before COVID-19. From 2016 to 2021, China’s annual growth rate for early-onset type 2 diabetes was 5.01%, while late-onset type 2 diabetes showed a declining trend with an annual change rate of -2.24%. This disparity may be related to China’s rapid digital economy development post-2015. By 2022, the digital economy accounted for 41.5% of GDP (30). This expansion has increased access to unhealthy foods, especially via the food delivery market among younger populations (31). A cross-sectional study among Chinese college students found that food delivery consumption was significantly associated with a preference for high-fat, high-sugar (HFHS) foods and with overweight and obesity (32). In contrast, elderly Chinese tend to adopt healthier lifestyles. A study from the Chinese Longitudinal Healthy Longevity Survey (CLHLS) conducted from 2005 to 2014 found that about 77.2% of the elderly improved their health behaviors (33).

The rising incidence of T2DM in India may be closely related to a conflict between religious beliefs, cultural habits, and the ongoing process of modernization (34–36). India, being a multi-religious and multi-cultural country, has religious practices such as those in Hinduism, Islam, and other faiths that promote dietary habits of moderation and vegetarianism, which traditionally help maintain a healthier diet (34, 36). However, with the increasing penetration of Western lifestyles, particularly among the younger generation who prefer high-sugar, high-fat foods, the influence of these traditional dietary habits has gradually weakened. Additionally, India has long faced ecological pressures, such as seasonal famines and increasing population density, which, over the course of long-term natural selection, have led to characteristics such as low birth weight, short stature, and low lean mass in its population, resulting in lower metabolic capacity (34, 35). This low metabolic capacity makes it more difficult for the population to cope with high metabolic loads, such as excess body fat, high-glycemic diets, and sedentary behavior, making them more susceptible to developing diabetes (34, 35). Therefore, despite the positive impact of India’s religious and cultural traditions on diet and health in the past, the shift in dietary patterns and lifestyles due to modernization is weakening the influence of these traditional practices, contributing to the rising diabetes burden.

Additionally, while new cases of early-onset type 2 diabetes are higher in India than in China, the ASIR is lower. Conversely, for late-onset type 2 diabetes, India has fewer cases but a higher ASIR than China. This is due to significant differences in population structures: China is experiencing significant aging and a declining birth rate, while India’s population is relatively young, with a lower proportion of people aged 65 and above (7). Overall, China’s type 2 diabetes burden is primarily influenced by aging and the accelerated growth in early-onset type 2 diabetes incidence, while India faces the dual challenges of rapid population growth and rising incidence rates for both early-onset and late-onset type 2 diabetes. Moreover, China and India have the highest number of diabetes cases globally and the largest number of undiagnosed diabetes patients, particularly among young people (37). Studies indicate that diabetes awareness among young people in China is significantly lower than among the elderly (38). These factors highlight the unique challenges and characteristics of the type 2 diabetes burden in China and India.

Despite the comprehensive analysis, our study has several limitations. First, the data derived from the GBD study relies heavily on the accuracy and completeness of national health information systems, which may vary significantly across countries in the Asia-Pacific region. Second, the GBD study uses modeling techniques and data imputation to address gaps, which could introduce uncertainties in the estimates. Additionally, the COVID-19 pandemic’s impact on healthcare access and health-seeking behaviors might have influenced the observed trends, but our study does not allow for a detailed examination of these factors. Another limitation is that we focused solely on incidence trends without analyzing changes in key risk factors, such as BMI, which should be explored in future studies. Finally, the long-term effects of COVID-19 on type 2 diabetes incidence in the Asia-Pacific region remain uncertain, necessitating ongoing surveillance and research. As the incidence of early-onset T2DM continues to rise in the Asia-Pacific region, future research should focus on investigating the impact of socio-cultural factors (such as dietary patterns, lifestyle modifications, and urbanization) on the onset and progression of the disease, while also implementing targeted interventions for high-risk populations. In particular, randomized controlled trials (RCTs) examining the efficacy of traditional antidiabetic therapies and emerging metabolism-related agents (such as tirzepatide, statins, and sacubitril/valsartan) in younger populations will be crucial (39). Optimizing the management of multiple metabolic risk factors with these therapies holds significant promise in reducing the risk of early-onset T2DM and its complications, ultimately improving long-term patient outcomes.

In conclusion, our study reveals a notably rapid increase in the incidence of early-onset type 2 diabetes in the Asia-Pacific region over the past ten years, particularly during the COVID-19 pandemic. Conversely, the incidence of late-onset type 2 diabetes has either increased or decreased in some countries. These findings underscore the urgent need for targeted public health interventions to address the rising burden of type 2 diabetes among younger populations in the Asia-Pacific region. Continuous monitoring and research are especially critical in countries with high burdens of early-onset type 2 diabetes, such as China, India, and Indonesia.

## Data Availability

Publicly available datasets were analyzed in this study. This data can be found here: https://vizhub.healthdata.org/gbd-results/.
