# Supplementary material for: Comparison of incidence trends of early-onset and late-onset type 2 diabetes in the Asia-Pacific region, 1990-2021: a join point regression analysis based on the global burden of disease study 2021
Source: Front Endocrinol (Lausanne). 2025 Feb 19;16:1466428. doi: 10.3389/fendo.2025.1466428 (PMC11879835; doi:10.3389/fendo.2025.1466428)
Supplement: Supplementary file 1 [file DataSheet1.docx]

Supplementary Material

**Contents**

**1.** **Methods for estimating non-fatal outcomes of type 2 diabetes -------------------------------------- 2**

**2. Supplementary Figures** **-------------------------------------------------------------------------------------- 5**

Supplementary **Figure 1. Distribution of countries in the WHO South-East Asia Region and Western Pacific Region** ------------------------------------------------------------------------------------------------------ 5

Supplementary **Figure 2. Proportion of new cases of early-onset type 2 diabetes in SEARO and WPRO countries, 1990-2021 -------------------------------------------------------------------------------**-----------**---- 6**

Supplementary **Figure** 3a: Joinpoint analysis of early-onset and late-onset type 2 diabetes ASIR in selected countries in the Asia-Pacific region ------------------------------------------------------------------- 7

Supplementary **Figure** 3b: Joinpoint analysis of early-onset and late-onset type 2 diabetes ASIR in selected countries in the WPRO --------------------------------------------------------------------------------- 8

**3. Supplementary tables ----------------------------------------------------------------------------------------- 9**

Supplementary **Table 1**: Number of incidence cases of early-onset and late-onset type 2 diabetes by region, country, and sex in 1990 and 2021----------------------------------------------------------------**-**----- 9

Supplementary **Table 2**. Net excess incidence number and rate of early-onset and late-onset type 2 diabetes by 5-year age groups in South-East Asia Region and Western Pacific Region for 2020 and 2021 --------------------------------------------------------------------------------------------------------------- 13

Supplementary **Table 3**. Net excess incidence number and rate of early-onset and late-onset type 2 diabetes at the country level in South-East Asia Region and Western Pacific Region for 2020 and 2021 ---------------------------------------------------------------------------------------------------------------------- 14

**4. Sex differences in ASIR and AAPC of early-onset and late-onset type 2 diabetes in SEARO and WPRO** ------------------------------------------------------------------------------------------------------- **17**

Supplementary **Figure** 4. ASIR of early-onset and late-onset type 2 diabetes by sex in the WHO South-East Asia Region and Western Pacific Region for the years 1990, 2010, and 2021--------------------- 17

**5. Incidence trends by 5-year age groups ------------------------------------------------------------------ 18**

Supplementary **Figure** 5: Incidence numbers and crude rates of type 2 diabetes across 5-year age groups in the WHO South-East Asia Region and Western Pacific Region for the years 1990, 2010, and 2021------------------------------------------------------------------------------------------------------------------------18

Supplementary **Table** 4: Incidence number and rates of type 2 diabetes by 5-year age groups in the South-East Asia Region and Western Pacific Region for the years 1990, 2010, and 2021-------------- 19

# Methods for estimating non-fatal outcomes of type 2 diabetes

## Diagnostic criteria for diabetes in GBD 2021

In GBD 2021, to comprehensively cover all available representative data, researchers accepted data from multiple blood glucose measurement methods. The definition and diagnostic criteria for diabetes were based on fasting plasma glucose (FPG), glycated hemoglobin A1c (HbA1c), oral glucose tolerance test (OGTT), and postprandial glucose test (PPG). However, data defining diabetes by random blood glucose or self-reported diabetes status were not accepted.

## Data inputs

**1.2.1 Total diabetes data**

Inputs In GBD 2021, the data for total diabetes came from various sources. Initially, 689 data sources were identified from the total diabetes model of GBD 2019, and 111 additional sources were identified through other channels, totaling 800 data sources. These sources were audited to assess their eligibility. During the evaluation, 210 data sources were excluded for various reasons, resulting in 590 data sources being included in the total diabetes model for GBD 2021. The data sources include the following categories:

- Estimates of diabetes in a representative population
- Estimates of mean FPG in a representative population
- Individual-level data of blood sugar from surveys
- **
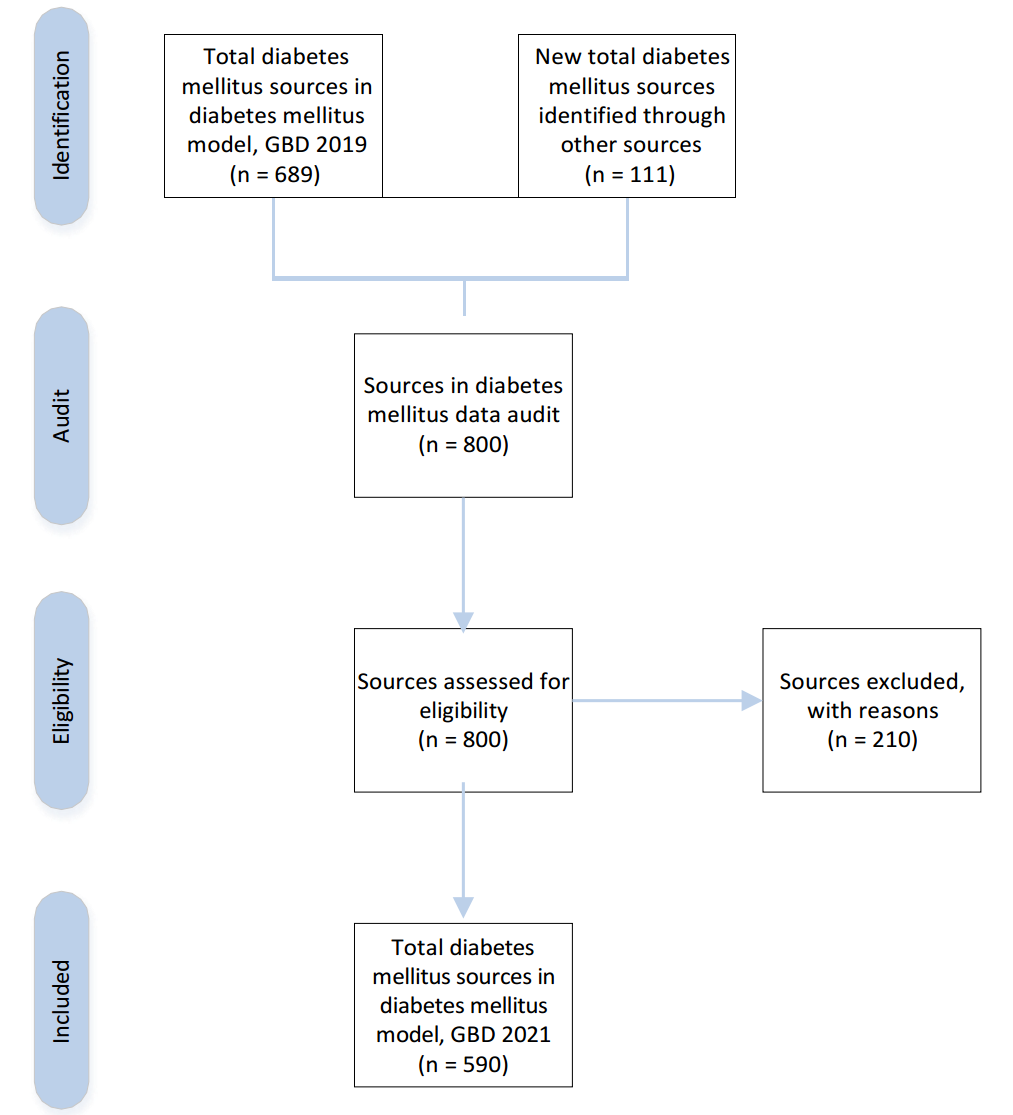
**Insurance claims data from the USA and Taiwan (province of China)

**Figure 1. Diagram of data sources in the GBD 2021 diabetes mellitus model**

**1.2.2 Type 1 diabetes data**

To include all available population-representative data on type 1 diabetes, GBD 2021 accepted data reporting type 1 diabetes, juvenile-onset diabetes, and childhood insulin-dependent diabetes. These data primarily came from the following sources:

- Published estimates of type 1 diabetes mellitus in a representative population
- Diabetes registry data

## Estimation process for non-fatal burden of type 2 diabetes

In GBD 2021, the estimation process for the non-fatal burden (prevalence and incidence) of type 2 diabetes involved three main steps:

1. Data collection and preparation for total diabetes: Age and sex stratification through MR-BRT (Meta-Regression—Bayesian, Regularised, Trimmed) analysis and standardization using Out-of-Dismod crosswalk methods. Modeling using Dismod-MR 2.1 (Disease Modelling - Meta Regression 2.1). Specific steps included handling outliers for small sample sizes, converting sources with average FPG data but lacking prevalence data to diabetes prevalence using ensemble distribution models, age and sex stratification, and bias adjustment using meta-regression models. To ensure accurate estimates for children under 15 years, total diabetes estimates were replaced with type 1 diabetes estimates, assuming type 2 diabetes does not occur before age 15.
2. Data collection and preparation for type 1 diabetes: Age and sex stratification and standardization through population-representative estimates of type 1 diabetes and diabetes registry data. Data for diabetes claims for individuals under 15 years were directly included in the model and modeled using Dismod-MR 2.1.
3.
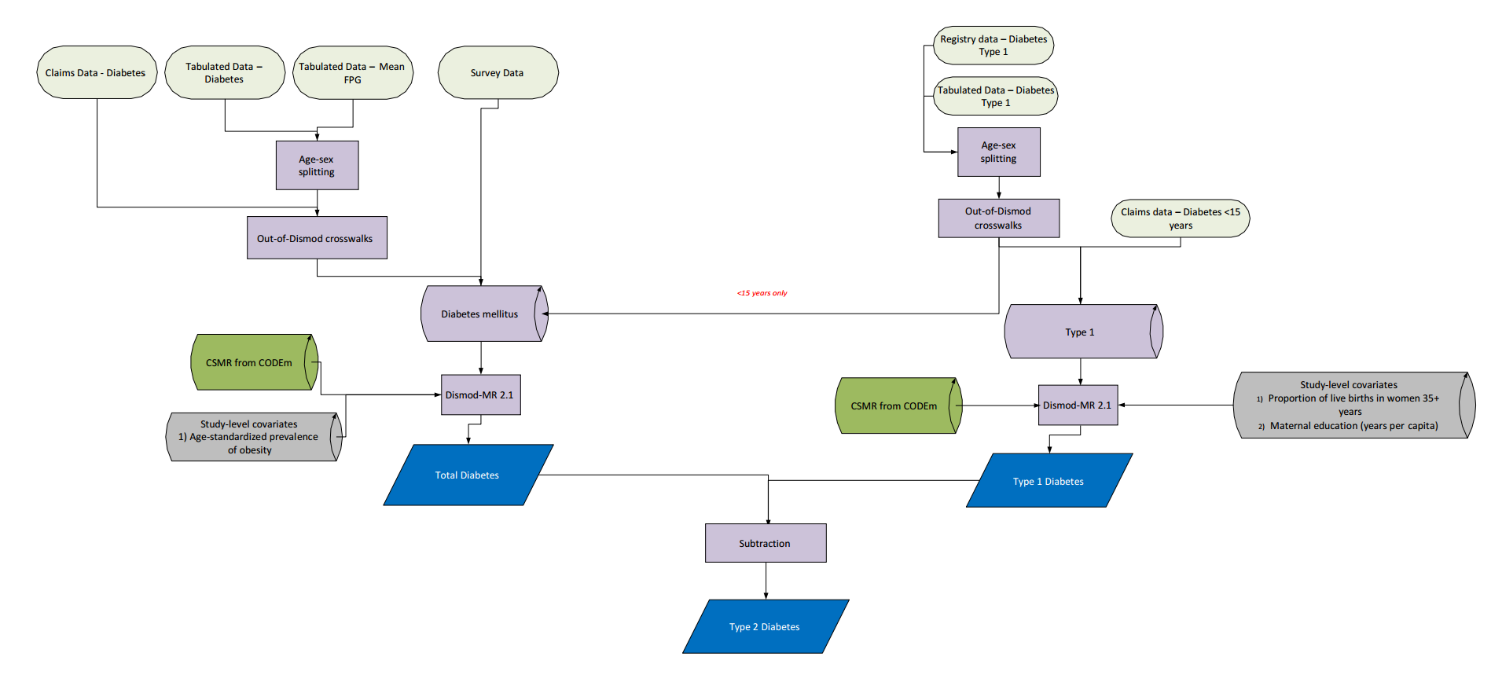
Estimation of type 2 diabetes: The non-fatal burden of type 2 diabetes was estimated by subtracting the type 1 diabetes estimates from the total diabetes estimates. Detailed data inputs, processing, and modeling strategies are elaborated in previous publications^1,2^.

**Figure 2. Calculating prevalence of diabetes mellitus (total, type 1, and type 2)**

References

1. Diseases GBD, Injuries C. Global incidence, prevalence, years lived with disability (YLDs), disability-adjusted life-years (DALYs), and healthy life expectancy (HALE) for 371 diseases and injuries in 204 countries and territories and 811 subnational locations, 1990-2021: a systematic analysis for the Global Burden of Disease Study 2021. Lancet. May 18 2024;403(10440):2133-2161. doi:10.1016/S0140-6736(24)00757-8

2. Collaborators GBDD. Global, regional, and national burden of diabetes from 1990 to 2021, with projections of prevalence to 2050: a systematic analysis for the Global Burden of Disease Study 2021. Lancet. Jul 15 2023;402(10397):203-234. doi:10.1016/S0140-6736(23)01301-6

#
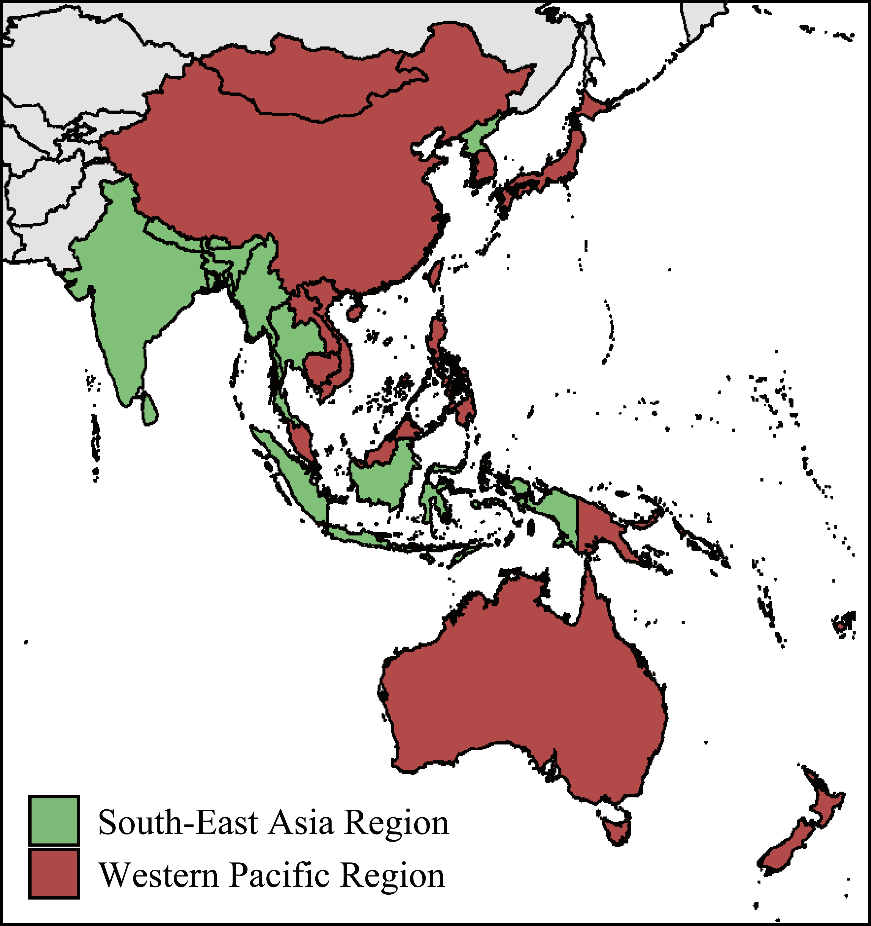
Supplementary Figures

**Supplementary Figure 1.** **Distribution of countries in the WHO South-East Asia Region and Western Pacific Region.** The South-East Asia Region (shown in green) includes 11 countries: Bangladesh, Bhutan, Democratic People's Republic of Korea, India, Indonesia, Maldives, Myanmar, Nepal, Sri Lanka, Thailand, and Timor-Leste. The Western Pacific Region (shown in red) includes 31 countries: American Samoa, Australia, Brunei Darussalam, Cambodia, China, Cook Islands, Fiji, Guam, Japan, Kiribati, Lao People's Democratic Republic, Malaysia, Marshall Islands, Micronesia (Federated States of), Mongolia, Nauru, New Zealand, Niue, Northern Mariana Islands, Palau, Papua New Guinea, Philippines, Republic of Korea, Samoa, Singapore, Solomon Islands, Tokelau, Tonga, Tuvalu, Vanuatu, and Viet Nam.


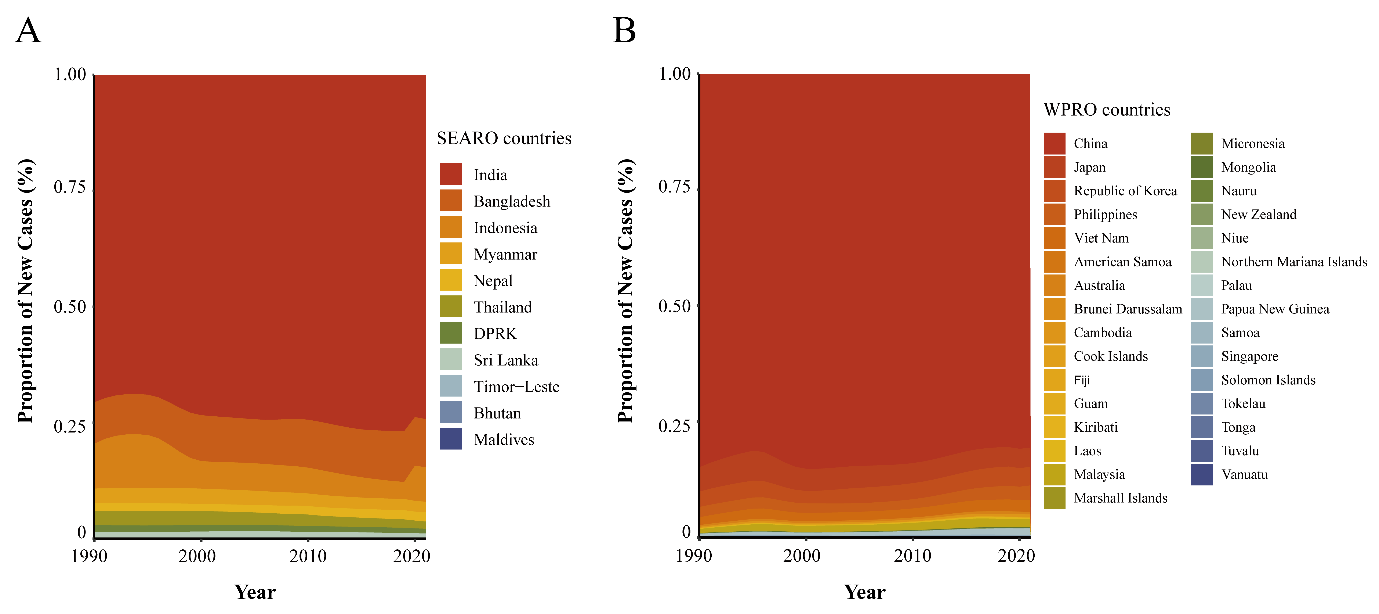


**Supplementary Figure 2. Proportion of new cases of early-onset type 2 diabetes in SEARO and WPRO countries, 1990-2021. (A) The proportion of new cases in the South-East Asia Region (SEARO) countries. (B) The proportion of new cases in the Western Pacific Region (WPRO) countries**


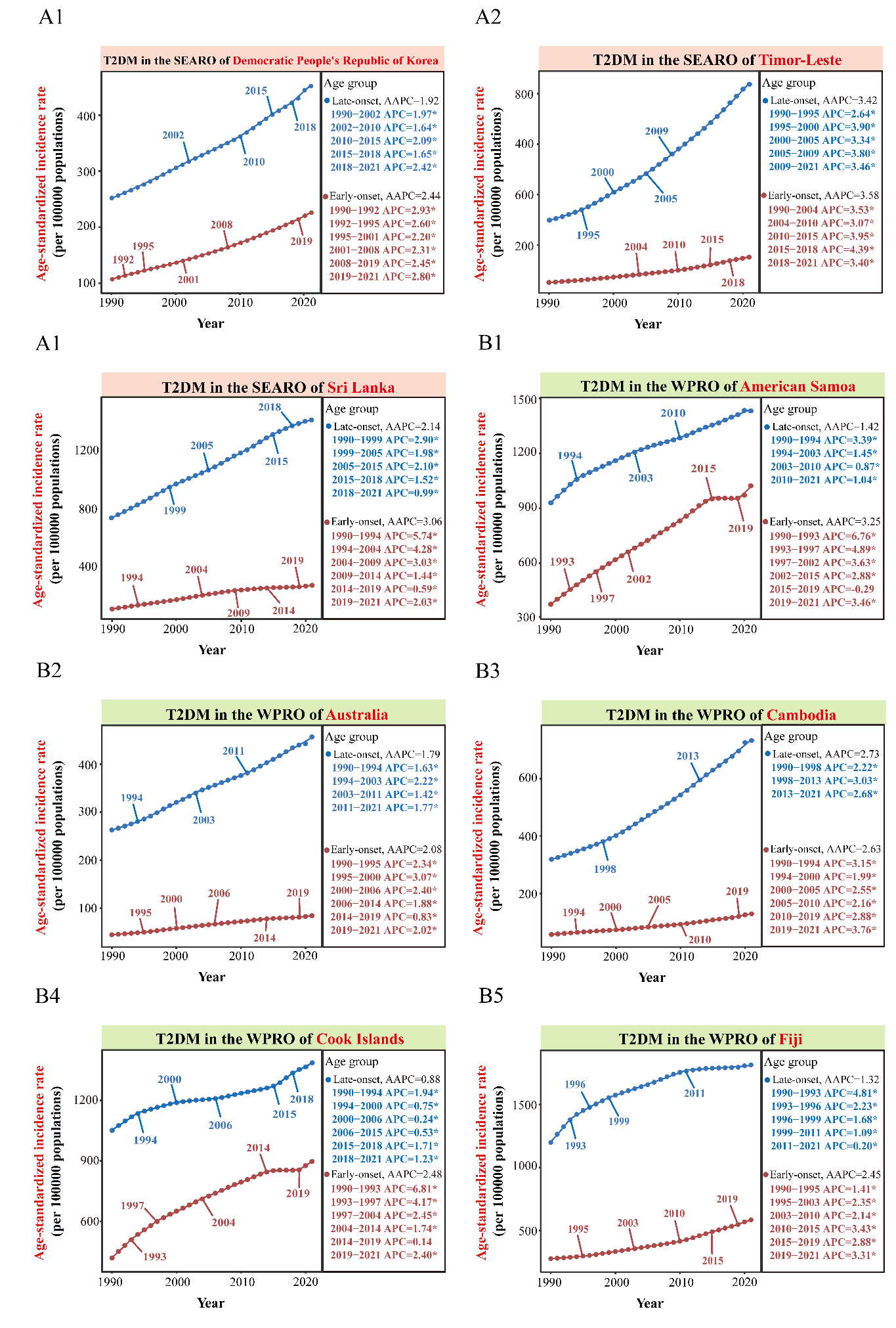
**Supplementary Figure 3a. Joinpoint analysis of early-onset and late-onset type 2 diabetes ASIR in selected countries in the Asia-Pacific region.** This figure presents joinpoint analysis for early-onset and late-onset type 2 diabetes mellitus age-standardized incidence rates (ASIR) in selected countries from the WHO South-East Asia Region (SEARO) and Western Pacific Region (WPRO). **(A1-A3)** Joinpoint analysis for SEARO countries with significant changes in early-onset type 2 diabetes ASIR during the COVID-19 pandemic. **(B1-B5)** Joinpoint analysis for WPRO countries with significant changes in early-onset type 2 diabetes ASIR during the COVID-19 pandemic. AAPC = average annual percent change. APC=annual percentage change. ^*^*P*<0.05 for significant APC.

**
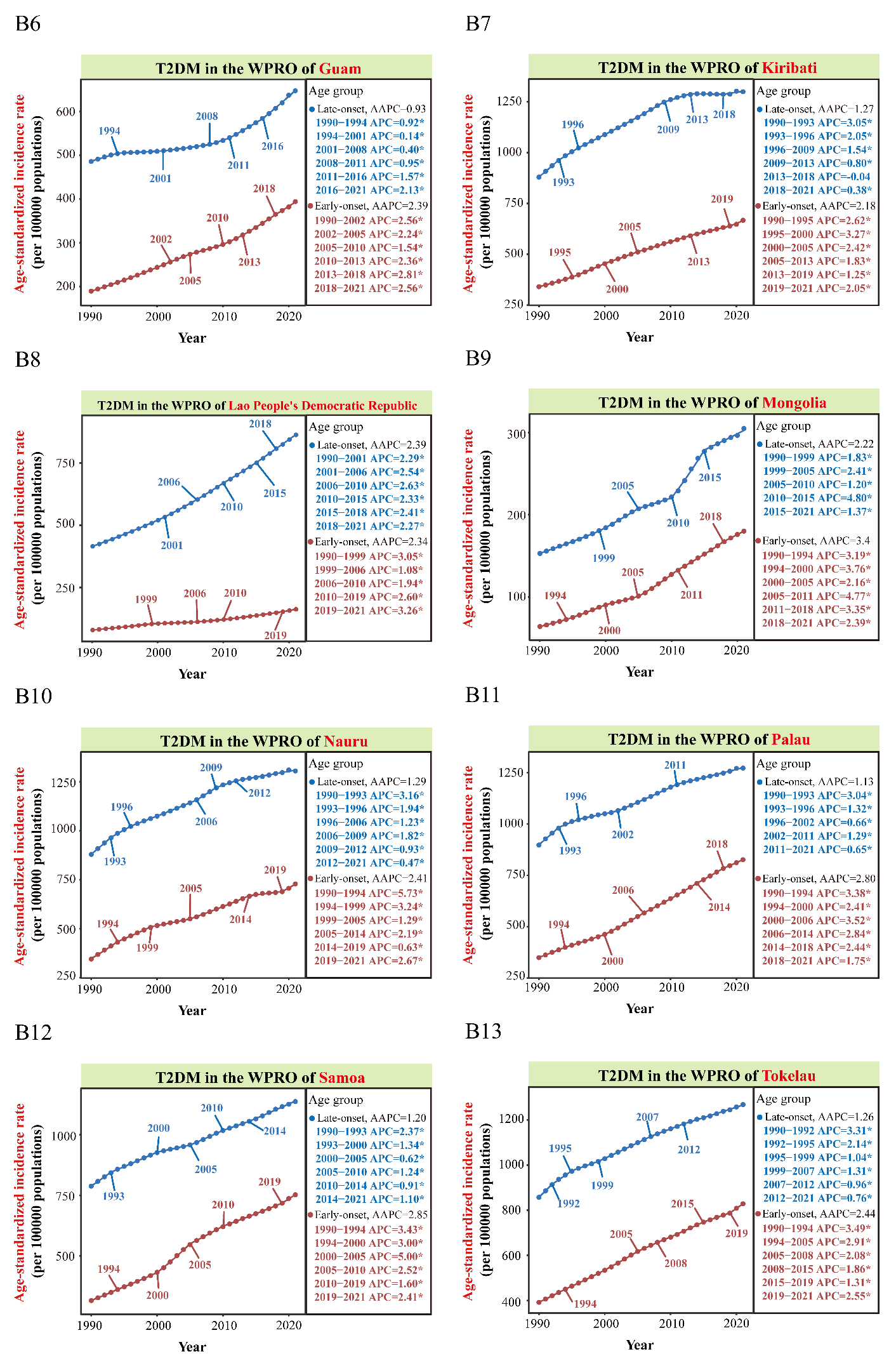
Supplementary Figure 3b. Joinpoint analysis of early-onset and late-onset type 2 diabetes ASIR in selected countries in the WPRO.** This figure presents joinpoint analysis for early-onset and late-onset type 2 diabetes mellitus age-standardized incidence rates (ASIR) in selected countries from WPRO region. (B6-B13) Joinpoint analysis for WPRO countries with significant changes in early-onset type 2 diabetes ASIR during the COVID-19 pandemic. AAPC = average annual percent change. APC=annual percentage change. ^*^*P*<0.05 for significant APC.

# Supplementary Tables

**Supplementary Table 1. Number of incidence cases of early-onset and late-onset type 2 diabetes by region, country, and sex in 1990 and 2021.**

| **Location/Sex** | **Number of Incidence Cases** | | | | |
| --- | --- | --- | --- | --- | --- |
|  | **Early-onset type 2 diabetes** | |  | **Late-onset type 2 diabetes** | |
|  | **1990** | **2021** |  | **1990** | **2021** |
| **South-East Asia Region** | 589336 (407574 to 796908) | 2149492 (1528994 to 2827339) |  | 921785 (684911 to 1206253) | 3748821 (2862758 to 4777629) |
| **Male** | 317444 (219479 to 428684) | 1202602 (864950 to 1580379) |  | 490601 (363963 to 643705) | 1903560 (1444042 to 2430330) |
| **Female** | 271892 (187416 to 368823) | 946889 (665426 to 1253707) |  | 431184 (322571 to 564619) | 1845261 (1412782 to 2349843) |
| Bangladesh | 52671 (37279 to 69619) | 222931 (161768 to 289251) |  | 54663 (42591 to 68442) | 270886 (204664 to 344662) |
| Bhutan | 276 (191 to 373) | 760 (550 to 1001) |  | 267 (196 to 348) | 1065 (804 to 1360) |
| Democratic People's Republic of Korea | 8937 (6116 to 12095) | 22210 (15987 to 29343) |  | 16391 (12119 to 21554) | 54077 (40100 to 68805) |
| India | 416149 (285580 to 569184) | 1594949 (1126106 to 2125075) |  | 582421 (417087 to 787259) | 2124372 (1543202 to 2817802) |
| Indonesia | 56831 (37470 to 78469) | 160627 (111566 to 214390) |  | 128596 (94462 to 169991) | 634867 (479396 to 814196) |
| Maldives | 53 (35 to 73) | 387 (273 to 529) |  | 153 (117 to 194) | 1027 (795 to 1279) |
| Myanmar | 18225 (12313 to 24971) | 49008 (35798 to 64304) |  | 47750 (35978 to 60591) | 201718 (159303 to 245847) |
| Nepal | 10090 (6951 to 13736) | 42591 (30555 to 56229) |  | 11148 (8065 to 14713) | 46341 (33803 to 60702) |
| Sri Lanka | 7638 (5072 to 10679) | 22084 (15269 to 29353) |  | 28277 (21739 to 35441) | 135912 (106649 to 165922) |
| Thailand | 18313 (12529 to 24809) | 33167 (23486 to 44037) |  | 51801 (39678 to 65185) | 275959 (216139 to 339656) |
| Timor-Leste | 154 (107 to 207) | 778 (552 to 1033) |  | 319 (249 to 399) | 2597 (2005 to 3272) |
| **Western Pacific Region** | 910605 (595911 to 1298106) | 1630010 (1158527 to 2167350) |  | 1527135 (1085320 to 2048897) | 4127438 (3043008 to 5379318) |
| **Male** | 526681 (344932 to 750266) | 973878 (696788 to 1288353) |  | 750258 (529859 to 1015769) | 2019188 (1471324 to 2660211) |
| **Female** | 383924 (249150 to 549774) | 656131 (459530 to 886272) |  | 776878 (554442 to 1034579) | 2108249 (1559735 to 2732012) |
| American Samoa | 74 (52 to 99) | 179 (127 to 240) |  | 84 (63 to 109) | 274 (200 to 353) |
| Australia | 3133 (2146 to 4390) | 8088 (5487 to 11529) |  | 16555 (12836 to 20234) | 55546 (41963 to 69343) |
| Brunei Darussalam | 184 (133 to 243) | 1015 (708 to 1382) |  | 364 (291 to 452) | 2415 (1826 to 3025) |
| Cambodia | 2062 (1394 to 2771) | 9447 (6742 to 12434) |  | 5215 (4052 to 6458) | 34232 (26790 to 42396) |
| China | 772813 (495354 to 1122229) | 1314733 (916726 to 1772161) |  | 1041417 (691746 to 1455478) | 2656753 (1825390 to 3627533) |
| Cook Islands | 31 (22 to 42) | 53 (38 to 70) |  | 49 (35 to 64) | 108 (81 to 137) |
| Fiji | 865 (616 to 1143) | 2101 (1542 to 2711) |  | 1681 (1308 to 2060) | 5646 (4478 to 6920) |
| Guam | 120 (86 to 158) | 218 (161 to 284) |  | 145 (111 to 184) | 435 (334 to 552) |
| Japan | 47806 (32688 to 65390) | 66989 (46309 to 92060) |  | 256758 (194277 to 330379) | 440617 (325532 to 575697) |
| Kiribati | 100 (71 to 132) | 327 (240 to 422) |  | 125 (94 to 158) | 409 (316 to 517) |
| Lao People's Democratic Republic | 1139 (789 to 1543) | 5141 (3680 to 6744) |  | 3126 (2406 to 3909) | 15517 (12080 to 19160) |
| Malaysia | 7761 (5188 to 10719) | 26282 (18265 to 35670) |  | 20579 (15796 to 25473) | 104606 (81814 to 129862) |
| Marshall Islands | 78 (54 to 104) | 271 (194 to 362) |  | 59 (43 to 78) | 230 (168 to 297) |
| Micronesia (Federated States of) | 110 (79 to 143) | 262 (192 to 343) |  | 126 (96 to 160) | 402 (309 to 502) |
| Mongolia | 525 (357 to 707) | 2380 (1731 to 3108) |  | 579 (437 to 744) | 3225 (2421 to 4148) |
| Nauru | 14 (10 to 18) | 33 (25 to 44) |  | 16 (12 to 21) | 34 (25 to 43) |
| New Zealand | 922 (540 to 1350) | 2345 (1712 to 3005) |  | 4395 (3226 to 5789) | 12234 (9971 to 14563) |
| Niue | 3 (2 to 4) | 5 (4 to 7) |  | 6 (4 to 8) | 9 (7 to 12) |
| Northern Mariana Islands | 49 (35 to 64) | 72 (53 to 94) |  | 43 (33 to 55) | 175 (133 to 220) |
| Palau | 24 (17 to 32) | 50 (36 to 65) |  | 33 (24 to 42) | 122 (91 to 153) |
| Papua New Guinea | 3910 (2821 to 5134) | 22947 (16950 to 29458) |  | 4325 (3277 to 5476) | 23791 (18061 to 29654) |
| Philippines | 20154 (13210 to 27963) | 50273 (34776 to 68323) |  | 55269 (41627 to 71267) | 205516 (157120 to 260742) |
| Republic of Korea | 30088 (22000 to 39197) | 65443 (47435 to 84731) |  | 58240 (45035 to 71387) | 280110 (221391 to 343889) |
| Samoa | 200 (141 to 265) | 599 (436 to 792) |  | 242 (181 to 311) | 637 (471 to 822) |
| Singapore | 2873 (1985 to 3898) | 6646 (4723 to 8909) |  | 5850 (4547 to 7268) | 22183 (17087 to 27738) |
| Solomon Islands | 235 (171 to 307) | 1136 (861 to 1445) |  | 273 (213 to 343) | 1387 (1103 to 1689) |
| Tokelau | 2 (2 to 3) | 4 (3 to 5) |  | 4 (3 to 5) | 6 (5 to 8) |
| Tonga | 94 (66 to 126) | 224 (161 to 294) |  | 163 (123 to 208) | 351 (269 to 442) |
| Tuvalu | 8 (6 to 10) | 24 (18 to 32) |  | 16 (12 to 20) | 39 (30 to 48) |
| Vanuatu | 116 (83 to 154) | 645 (474 to 832) |  | 130 (98 to 167) | 679 (517 to 856) |
| Viet Nam | 15358 (10420 to 20791) | 42551 (31042 to 55388) |  | 51544 (39796 to 63859) | 260641 (212025 to 314182) |

Data in parentheses are 95% uncertainty intervals (UIs) for the number of incidence cases.

**Supplementary Table 2. Net excess incidence number and rate of early-onset and late-onset type 2 diabetes by 5-year age groups in South-East Asia Region and Western Pacific Region for 2020 and 2021.**

| **Age group** | **South-East Asia Region** | | | | |  | **Western Pacific Region** | | | | |
| --- | --- | --- | --- | --- | --- | --- | --- | --- | --- | --- | --- |
|  | **Net excess number of incidence** | |  | **Net excess incidence rates**  **(per 100,000)** | |  | **Net excess number of incidence** | |  | **Net excess incidence rates**  **(per 100,000)** | |
|  | **2020** | **2021** |  | **2020** | **2021** |  | **2020** | **2021** |  | **2020** | **2021** |
| Early-onset T2DM | 83599 | 117344 |  | 9.8 | 13.6 |  | 69931 | 75300 |  | 14.2 | 15.3 |
| 15 to 19 | 11142 | 15920 |  | 5.9 | 8.4 |  | 21665 | 24676 |  | 20.2 | 22.7 |
| 20 to 24 | 25479 | 34203 |  | 13.8 | 18.4 |  | 27566 | 26801 |  | 25.0 | 24.8 |
| 25 to 29 | 27384 | 39046 |  | 15.8 | 22.2 |  | -21550 | -20179 |  | -16.6 | -16.4 |
| 30 to 34 | 39978 | 51925 |  | 24.5 | 31.5 |  | -41305 | -40456 |  | -25.7 | -25.6 |
| 35 to 39 | 59428 | 71231 |  | 39.3 | 46.3 |  | -23837 | -28408 |  | -17.7 | -20.1 |
| Late-onset T2DM | 147274 | 216657 |  | 18.1 | 27.6 |  | -140650 | -142633 |  | -13.5 | -12.7 |
| 40 to 44 | 61833 | 75986 |  | 46.2 | 55.7 |  | -7339 | -9638 |  | -5.8 | -7.7 |
| 45 to 49 | 53966 | 69794 |  | 45.6 | 58.0 |  | -3464 | -5502 |  | -2.3 | -3.8 |
| 50 to 54 | 43785 | 57427 |  | 42.8 | 54.8 |  | -12030 | -6282 |  | -7.9 | -4.1 |
| 55 to 59 | 36306 | 45482 |  | 42.4 | 51.7 |  | -17539 | -21764 |  | -13.6 | -15.9 |
| 60 to 64 | 14607 | 23178 |  | 20.4 | 31.4 |  | 12100 | 17451 |  | 12.4 | 18.0 |
| 65 to 69 | -13153 | -3831 |  | -23.3 | -6.6 |  | -1684 | -5561 |  | -1.8 | -5.7 |
| 70 to 74 | -35392 | -36380 |  | -91.2 | -89.6 |  | -6005 | -8965 |  | -8.9 | -12.6 |
| 75 to 79 | -37106 | -41433 |  | -149.3 | -162.5 |  | -10732 | -13252 |  | -24.4 | -29.2 |
| 80 to 84 | -13929 | -15789 |  | -97.1 | -108.1 |  | -5674 | -6258 |  | -20.1 | -21.6 |
| 85 to 89 | -1237 | -1409 |  | -20.0 | -22.0 |  | -414 | -467 |  | -2.8 | -3.1 |
| 90 to 94 | 12 | 8 |  | 0.6 | 0.4 |  | 135 | 158 |  | 2.5 | 2.8 |
| 95 plus | 5 | 4 |  | 1.1 | 0.9 |  | 52 | 64 |  | 3.3 | 3.7 |

The net excess incidence rates for early-onset and late-onset type 2 diabetes are age-standardized (ASIR), while the rates for each 5-year age group are crude incidence rates.

**Supplementary Table 3. Net excess incidence number and rate of early-onset and late-onset type 2 diabetes at the country level in South-East Asia Region and Western Pacific Region for 2020 and 2021.**

| **Location** | **Early-onset type 2 diabetes** | | | | |  | **Late-onset type 2 diabetes** | | | | |
| --- | --- | --- | --- | --- | --- | --- | --- | --- | --- | --- | --- |
|  | **Net excess number of incidence** | |  | **Net excess ASIR**  **(per 100,000)** | |  | **Net excess number of incidence** | |  | **Net excess ASIR**  **(per 100,000)** | |
|  | **2020** | **2021** |  | **2020** | **2021** |  | **2020** | **2021** |  | **2020** | **2021** |
| **South-East Asia Region** |  |  |  |  |  |  |  |  |  |  |  |
| Bangladesh | -3779 | -7231 |  | -5.4 | -10.3 |  | 10775 | 15533 |  | 20.0 | 28.7 |
| Bhutan | -10 | -2 |  | -2.5 | 0.0 |  | -12 | 0 |  | -6.4 | -1.5 |
| Democratic People's Republic of Korea | 165 | 180 |  | 2.0 | 2.4 |  | 1244 | 1309 |  | 10.4 | 10.3 |
| India | -2539 | 28189 |  | -0.5 | 4.6 |  | 63186 | 116845 |  | 13.7 | 25.0 |
| Indonesia | 85772 | 91125 |  | 74.5 | 78.6 |  | 31125 | 29417 |  | -12.5 | -13.8 |
| Maldives | 3 | 1 |  | 1.7 | 0.9 |  | 4 | 2 |  | -1.9 | -6.9 |
| Myanmar | 686 | 642 |  | 3.2 | 3.1 |  | 20208 | 21126 |  | 108.6 | 109.2 |
| Nepal | 162 | 1257 |  | 2.1 | 10.2 |  | 1300 | 1899 |  | 14.4 | 20.5 |
| Sri Lanka | 381 | 720 |  | 4.5 | 8.6 |  | 4715 | 4142 |  | 53.9 | 48.5 |
| Thailand | 46 | -1630 |  | 0.1 | -7.2 |  | 17716 | 27469 |  | 47.1 | 69.8 |
| Timor-Leste | -2 | -11 |  | -0.5 | -1.9 |  | -4 | -37 |  | -3.8 | -13.6 |
| **Western Pacific Region** |  |  |  |  |  |  |  |  |  |  |  |
| American Samoa | -7 | -2 |  | -39.9 | -6.2 |  | 0 | -5 |  | -2.0 | -21.6 |
| Australia | 65 | 162 |  | 0.6 | 1.6 |  | 1320 | 2375 |  | 13.6 | 22.5 |
| Brunei Darussalam | -2 | 6 |  | 0.6 | 4.0 |  | -30 | -46 |  | -6.2 | -14.6 |
| Cambodia | 176 | 185 |  | 2.6 | 2.7 |  | 2307 | 2139 |  | 44.6 | 38.3 |
| China | 64003 | 61280 |  | 19.0 | 18.8 |  | -77380 | -71728 |  | -9.1 | -7.5 |
| Cook Islands | -2 | -1 |  | -23.3 | -14.3 |  | -1 | -2 |  | -2.3 | 0.1 |
| Fiji | 11 | 10 |  | 3.0 | 3.1 |  | 114 | 104 |  | 14.9 | 14.4 |
| Guam | -1 | 0 |  | -0.2 | 1.8 |  | 3 | 0 |  | 5.6 | 3.2 |
| Japan | 3700 | 4138 |  | 10.3 | 11.4 |  | -16123 | -23164 |  | -29.8 | -40.3 |
| Kiribati | -5 | -2 |  | -9.7 | -1.6 |  | -2 | -5 |  | -29.1 | -37.6 |
| Lao People's Democratic Republic | 27 | 61 |  | 1.0 | 2.1 |  | 290 | 370 |  | 8.3 | 8.9 |
| Malaysia | 1175 | 770 |  | 8.6 | 5.8 |  | 8359 | 11927 |  | 82.2 | 114.7 |
| Marshall Islands | -14 | -14 |  | -55.0 | -55.1 |  | -4 | -5 |  | -22.0 | -28.4 |
| Micronesia (Federated States of) | -6 | -13 |  | -13.4 | -26.8 |  | -11 | -12 |  | -49.0 | -50.0 |
| Mongolia | -32 | -54 |  | -2.2 | -3.7 |  | -45 | -16 |  | -7.9 | -5.6 |
| Nauru | -1 | -1 |  | -26.1 | -19.2 |  | -1 | -1 |  | -13.7 | -32.5 |
| New Zealand | -8 | -26 |  | -0.2 | -0.9 |  | 45 | 3 |  | 2.2 | 0.3 |
| Niue | -1 | -1 |  | -191.5 | -175.9 |  | -1 | -1 |  | -72.7 | -93.5 |
| Northern Mariana Islands | 0 | 0 |  | 3.2 | 3.4 |  | 1 | 0 |  | 5.8 | 5.6 |
| Palau | -2 | -3 |  | -27.3 | -36.0 |  | -4 | -7 |  | -53.2 | -75.2 |
| Papua New Guinea | 759 | 999 |  | 15.9 | 21.8 |  | -559 | -607 |  | -23.3 | -25.5 |
| Philippines | -145 | 1533 |  | -0.4 | 3.3 |  | -243 | 2221 |  | -3.7 | 2.4 |
| Republic of Korea | 150 | 338 |  | -0.1 | 1.8 |  | -1871 | -1829 |  | -1.0 | -1.1 |
| Samoa | 2 | 4 |  | 4.8 | 8.2 |  | -4 | -7 |  | -9.7 | -12.9 |
| Singapore | -13 | 5 |  | 0.5 | 1.2 |  | -715 | -379 |  | -24.3 | -14.0 |
| Solomon Islands | -21 | -22 |  | -7.2 | -7.1 |  | -11 | -26 |  | -28.1 | -40.7 |
| Tokelau | -1 | -2 |  | -219.5 | -225.4 |  | 1 | 1 |  | 214.5 | 228.7 |
| Tonga | -2 | -2 |  | -3.7 | -2.4 |  | -1 | -4 |  | -6.6 | -14.9 |
| Tuvalu | -1 | -1 |  | -5.8 | -2.6 |  | -1 | -2 |  | -24.0 | -34.6 |
| Vanuatu | -4 | -1 |  | -2.7 | 0.7 |  | 0 | -3 |  | -7.4 | -12.5 |
| Viet Nam | -372 | 1824 |  | -0.8 | 4.3 |  | 25418 | 33743 |  | 66.7 | 83.3 |

ASIR = age-standardized incidence rate.

# Sex differences in ASIR and AAPC of early-onset and late-onset type 2 diabetes in SEARO and WPRO

From a sex perspective, in SEARO, males consistently had higher ASIR for both early-onset and late-onset type 2 diabetes in 1990, 2010, and 2021 compared to females. However, the rate of increase differed slightly: males had higher AAPC for early-onset type 2 diabetes ASIR during both 1990-2010 and 2010-2021, while for late-onset type 2 diabetes, females had higher AAPC compared to males during the same periods (Fig. 1; Table 1; Fig. S3).

In WPRO, males had higher ASIR for early-onset type 2 diabetes in 1990, 2010, and 2021, while females had higher ASIR for late-onset type 2 diabetes in these years. During 1990-2010, males exhibited higher AAPC for ASIR of both early-onset and late-onset type 2 diabetes compared to females. However, in the most recent decade (2010-2021), females showed slightly higher AAPC of both early-onset and late-onset type 2 diabetes compared to males (Fig. 1; Table 1; Fig. S3).


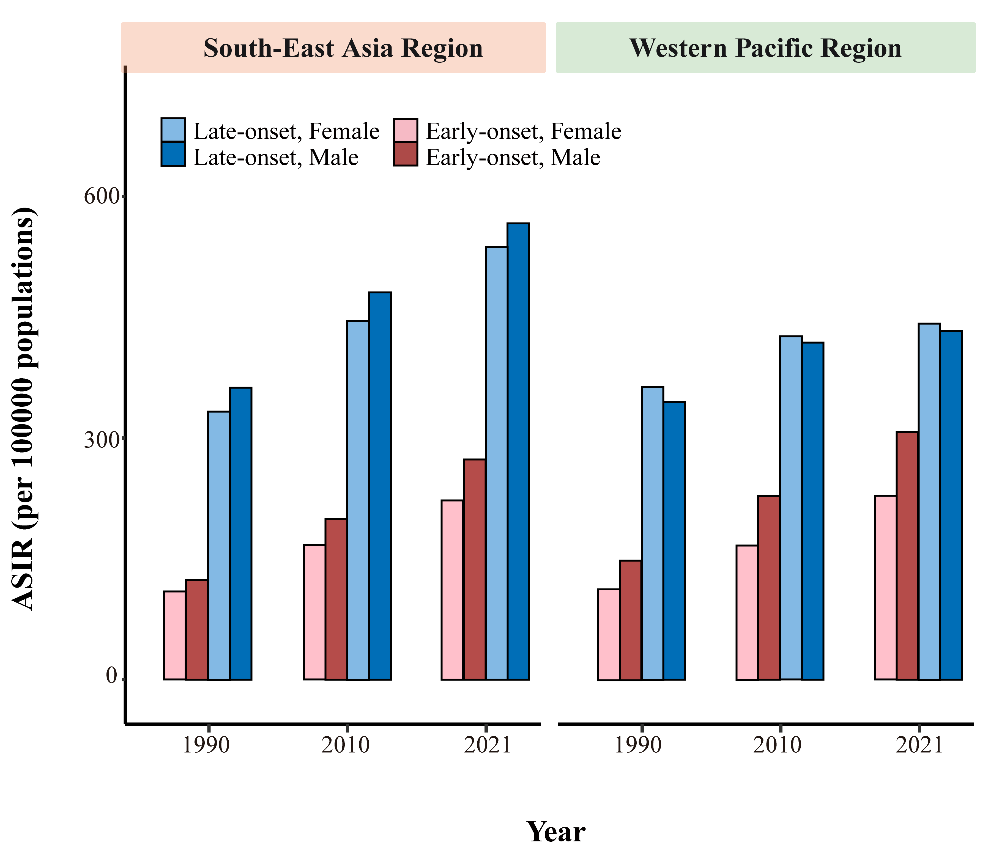


**Supplementary Figure 4. ASIR of early-onset and late-onset type 2 diabetes by sex in the WHO South-East Asia Region and Western Pacific Region for the years 1990, 2010, and 2021**. ASIR = age-standardized incidence rates.

# Incidence trends by 5-year age groups

In the Asia-Pacific region, including SEARO and WPRO, the number of type 2 diabetes cases across most 5-year age groups increased progressively from 1990 to 2010 and further to 2021. In SEARO, the highest number of type 2 diabetes cases in 2021 was in the 40 to 44 age group, reaching 0.63 million cases (95% UI: 0.49 million to 0.78 million). In WPRO, the highest number of type 2 diabetes cases in 2021 was in the 50 to 54 age group, with 0.81 million cases (95% UI: 0.61 million to 1.04 million) (Fig. S4; Table S4).

For incidence rates, the peak age group in SEARO shifted downward over time, from 630.9 per 100,000 (95% UI: 501.9 to 792.8) in the 75 to 79 age group in 1990 to 706.6 per 100,000 (95% UI: 552.1 to 866.9) in the 65 to 69 age group in 2021. Conversely, in WPRO, the peak incidence age group shifted upward, from 468.0 per 100,000 (95% UI: 333.2 to 617.7) in the 55 to 59 age group in 1990 to 608.9 per 100,000 (95% UI: 452.4 to 772.2) in the 60 to 64 age group in 2021. Notably, in WPRO, children and adolescents (ages 15 to 24) consistently exhibited a secondary peak in type 2 diabetes incidence rates across the years, surpassing the rates observed in young adults aged 25 to 39 (Fig. S4; Table S4).


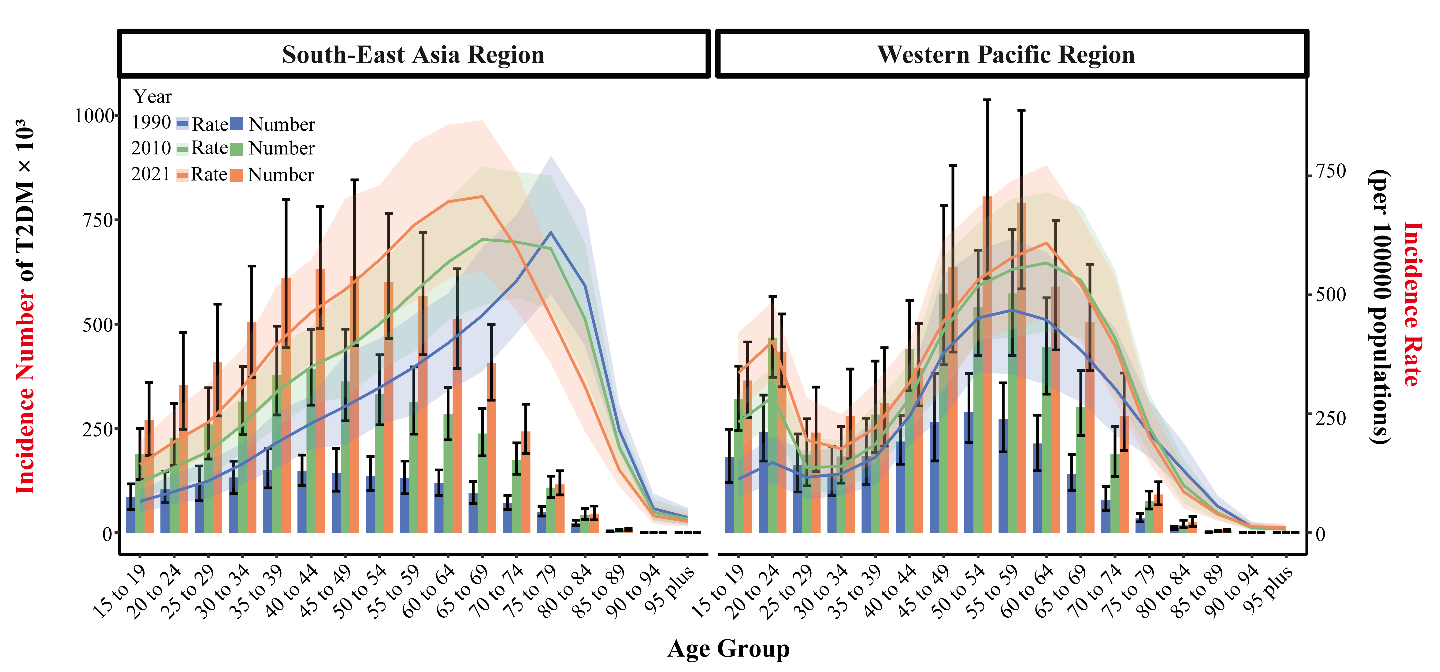


**Supplementary Figure 5. Incidence numbers and crude rates of type 2 diabetes across 5-year age groups in the WHO South-East Asia Region and Western Pacific Region for the years 1990, 2010, and 2021.** Bars represent incidence numbers, and lines depict crude incidence rates. Shaded areas and error bars indicate the 95% uncertainty intervals (UIs).

**Supplementary Table 4. Incidence number and rates of type 2 diabetes by 5-year age groups in the South-East Asia Region and Western Pacific Region for the years 1990, 2010, and 2021.**

| **Location** | **Number of Incidence** | | |  | **Incidence Rate (per 100,000)** | | |
| --- | --- | --- | --- | --- | --- | --- | --- |
| **Age groups** | **1990** | **2010** | **2021** |  | **1990** | **2010** | **2021** |
| **SEARO** |  |  |  |  |  |  |  |
| 15 to 19 | 86418  (55664 to 117184) | 188067  (127430 to 250712) | 270761  (185304 to 360694) |  | 66.0  (42.5 to 89.5) | 105.7  (71.6 to 140.9) | 143.7  (98.3 to 191.4) |
| 20 to 24 | 104082  (72620 to 146617) | 227790  (160837 to 310305) | 354598  (248050 to 480540) |  | 86.7  (60.5 to 122.1) | 137.8  (97.3 to 187.7) | 191.2  (133.7 to 259.1) |
| 25 to 29 | 115902  (77664 to 160075) | 259555  (176838 to 347695) | 408570  (280107 to 548151) |  | 109.0  (73.0 to 150.5) | 170.6  (116.3 to 228.6) | 232.7  (159.6 to 312.2) |
| 30 to 34 | 132443  (93915 to 171668) | 314304  (235114 to 398646) | 505193  (371948 to 639552) |  | 144.9  (102.7 to 187.8) | 227.1  (169.9 to 288.1) | 306.0  (225.3 to 387.4) |
| 35 to 39 | 150491  (107711 to 201365) | 377558  (282277 to 495443) | 610369  (443584 to 798401) |  | 189.7  (135.8 to 253.8) | 297.0  (222.1 to 389.8) | 396.5  (288.2 to 518.7) |
| 40 to 44 | 148714  (113612 to 186456) | 391795  (305936 to 486675) | 631836  (489821 to 781972) |  | 230.0  (175.7 to 288.4) | 348.7  (272.3 to 433.1) | 463.0  (358.9 to 573.0) |
| 45 to 49 | 143045  (99889 to 201789) | 362560  (268406 to 488067) | 615058  (449404 to 845802) |  | 265.7  (185.5 to 374.8) | 384.3  (284.5 to 517.4) | 511.4  (373.7 to 703.3) |
| 50 to 54 | 136860  (101739 to 183205) | 332882  (258879 to 426811) | 600697  (466045 to 764559) |  | 305.0  (226.8 to 408.3) | 438.6  (341.1 to 562.3) | 573.6  (445.0 to 730.1) |
| 55 to 59 | 130841  (93273 to 172102) | 313782  (236604 to 397547) | 568174  (426908 to 719663) |  | 348.1  (248.1 to 457.8) | 505.0  (380.8 to 639.8) | 646.3  (485.6 to 818.6) |
| 60 to 64 | 119401  (89414 to 150607) | 283640  (222952 to 349080) | 513093  (394084 to 632563) |  | 398.6  (298.5 to 502.8) | 568.6  (446.9 to 699.7) | 695.6  (534.3 to 857.6) |
| 65 to 69 | 94298  (69955 to 123798) | 237934  (184411 to 297212) | 407069  (318035 to 499420) |  | 456.8  (338.9 to 599.7) | 616.5  (477.8 to 770.1) | 706.6  (552.1 to 866.9) |
| 70 to 74 | 71231  (56427 to 89658) | 173823  (140624 to 215625) | 242811  (189823 to 308299) |  | 529.7  (419.6 to 666.7) | 611.0  (494.3 to 758.0) | 598.3  (467.7 to 759.7) |
| 75 to 79 | 50436  (40126 to 63378) | 108001  (84734 to 135824) | 115958  (90881 to 148554) |  | 630.9  (501.9 to 792.8) | 597.2  (468.5 to 751.1) | 454.9  (356.5 to 582.7) |
| 80 to 84 | 22995  (17403 to 30205) | 42809  (31548 to 58038) | 44829  (31085 to 64106) |  | 518.9  (392.7 to 681.6) | 448.9  (330.8 to 608.6) | 307.0  (212.8 to 438.9) |
| 85 to 89 | 3708  (2936 to 4640) | 6731  (5157 to 8791) | 8454  (6223 to 11289) |  | 215.5  (170.6 to 269.7) | 179.6  (137.6 to 234.6) | 131.9  (97.1 to 176.2) |
| 90 to 94 | 226  (121 to 365) | 527  (289 to 860) | 728  (387 to 1212) |  | 50.7  (27.1 to 82.0) | 43.1  (23.6 to 70.2) | 35.9  (19.1 to 59.7) |
| 95 plus | 31  (16 plus 50) | 92  (49 to 151) | 114  (61 to 190) |  | 32.1  (17.2 to 52.8) | 29.4  (15.7 to 47.9) | 24.0  (12.9 to 40.0) |
| **WPRO** |  |  |  |  |  |  |  |
| 15 to 19 | 181872  (120812 to 248384) | 319766  (244873 to 399539) | 364949  (276716 to 457342) |  | 112.6  (74.8 to 153.7) | 232.5  (178.1 to 290.6) | 335.5  (254.4 to 420.4) |
| 20 to 24 | 241333  (171873 to 330113) | 467521  (372758 to 566056) | 434303  (349727 to 524466) |  | 147.6  (105.1 to 201.9) | 285.3  (227.5 to 345.5) | 402.3  (324.0 to 485.8) |
| 25 to 29 | 162065  (97910 to 237019) | 187384  (112319 to 273025) | 240184  (146829 to 349274) |  | 116.5  (70.4 to 170.4) | 137.2  (82.2 to 199.9) | 194.8  (119.1 to 283.3) |
| 30 to 34 | 142172  (89570 to 207735) | 182784  (119114 to 255046) | 279127  (177321 to 392558) |  | 123.9  (78.0 to 181.0) | 139.4  (90.9 to 194.6) | 176.9  (112.4 to 248.8) |
| 35 to 39 | 183163  (115745 to 274854) | 283466  (192378 to 411595) | 311446  (207933 to 443711) |  | 158.7  (100.3 to 238.1) | 186.3  (126.4 to 270.5) | 220.6  (147.3 to 314.3) |
| 40 to 44 | 218910  (164569 to 281593) | 441026  (340167 to 556310) | 394845  (304119 to 502160) |  | 245.1  (184.3 to 315.3) | 282.2  (217.7 to 356.0) | 315.4  (242.9 to 401.1) |
| 45 to 49 | 265264  (172301 to 381929) | 573154  (402766 to 783664) | 636211  (433320 to 880247) |  | 378.4  (245.8 to 544.9) | 425.9  (299.3 to 582.3) | 445.0  (303.1 to 615.7) |
| 50 to 54 | 289635  (216547 to 381714) | 541597  (424247 to 677286) | 806017  (609212 to 1037608) |  | 451.4  (337.5 to 594.8) | 518.7  (406.3 to 648.6) | 531.7  (401.9 to 684.4) |
| 55 to 59 | 272319  (193880 to 359465) | 573647  (424848 to 727126) | 791245  (584608 to 1012751) |  | 468.0  (333.2 to 617.7) | 553.5  (409.9 to 701.5) | 578.4  (427.4 to 740.4) |
| 60 to 64 | 214130  (148711 to 281912) | 445996  (332310 to 563178) | 590163  (438418 to 748419) |  | 447.5  (310.8 to 589.2) | 567.0  (422.4 to 715.9) | 608.9  (452.4 to 772.2) |
| 65 to 69 | 141554  (101196 to 187314) | 301792  (232828 to 389385) | 505079  (389554 to 643686) |  | 384.0  (274.5 to 508.1) | 531.1  (409.8 to 685.3) | 521.2  (402.0 to 664.2) |
| 70 to 74 | 78110  (53206 to 110852) | 188583  (135531 to 254731) | 280276  (197618 to 383019) |  | 303.5  (206.8 to 430.8) | 408.9  (293.9 to 552.4) | 392.4  (276.7 to 536.2) |
| 75 to 79 | 34506  (26274 to 45790) | 75742  (57198 to 99992) | 91772  (66999 to 122210) |  | 209.3  (159.4 to 277.8) | 221.5  (167.3 to 292.4) | 201.9  (147.4 to 268.9) |
| 80 to 84 | 10912  (7360 to 15791) | 19537  (12084 to 30169) | 24994  (14851 to 39042) |  | 131.0  (88.4 to 189.6) | 100.3  (62.1 to 154.9) | 86.3  (51.3 to 134.8) |
| 85 to 89 | 1699  (1228 to 2358) | 3497  (2360 to 5080) | 5923  (3850 to 8599) |  | 55.9  (40.4 to 77.6) | 41.2  (27.8 to 59.8) | 38.8  (25.2 to 56.3) |
| 90 to 94 | 86  (41 to 159) | 280  (133 to 503) | 725  (360 to 1255) |  | 12.1  (5.8 to 22.3) | 10.5  (5.0 to 18.8) | 12.9  (6.4 to 22.4) |
| 95 plus | 11  (7 to 19) | 54  (24 to 96) | 188  (99 to 320) |  | 8.0  (4.9 to 14.4) | 7.4  (3.2 to 13.1) | 10.9  (5.8 to 18.7) |

Data in parentheses are 95% uncertainty intervals (UIs). SEARO = South-East Asia Region. WPRO = Western Pacific Region.
